# Modeling and Optimization of Transition Metal-Catalyzed Peracetic Acid Oxidation for Advanced Polishing of Biologically Treated Leachate

**DOI:** 10.1021/acsomega.6c03464

**Published:** 2026-06-08

**Authors:** Emre Ünal, Ceyda Tunçak, Rabia Nefise Yılmaz, Emine Can-Güven, Senem Yazici Guvenc, Gamze Varank

**Affiliations:** Faculty of Civil Engineering, Department of Environmental Engineering, 52999 Yildiz Technical University, Istanbul 34220, Türkiye

## Abstract

The treatment of landfill leachate is a significant environmental challenge because of its high concentration of resistant organic pollutants that are difficult to remove using conventional methods. This study aimed to remove resistant organic matter from the membrane bioreactor effluent (MBR) of leachate through the use of advanced oxidation processes. The catalytic effect of transition metals (Fe^2+^, Zn^2+^, Cu^2+^, Mn^2+^, Ni^2+^, and Co^2+^) was evaluated, where peracetic acid (PAA) was used as the oxidant. Among the applied processes, the highest pollutant removal was obtained by Fe^2+^/PAA, Co^2+^/PAA, and Mn^2+^/PAA processes. The Box–Behnken design was used for parameter optimization of these processes. The initial pH, catalyst dose, and oxidant dose were independent variables, whereas COD and UV_254_ removal efficiencies were system responses. Regression models describing COD and UV_254_ removal through Fe^2+^/PAA, Co^2+^/PAA, and Mn^2+^/PAA processes were developed, and the data obtained were analyzed by analysis of variance. Under the optimum conditions for the Fe^2+^/PAA process (pH: 3.13, Fe^2+^: 0.233 mM, PAA: 2.25 mM), the COD and UV_254_ removal were 86.1% and 87.5%, respectively. For the Co^2+^/PAA process under optimum conditions (pH: 3.93, Co^2+^: 0.298 mM, PAA: 2.16 mM), the COD and UV_254_ removal were 80.2% and 86.0%, respectively. For the Mn^2+^/PAA process under optimum conditions (pH: 3.12, Mn^2+^: 0.299 mM, and PAA: 2.22 mM), the COD and UV_254_ removal were 84.0% and 84.8%, respectively. These results showed that the optimized transition-metal-catalyzed PAA processes provide a highly efficient approach for the advanced treatment of real leachate MBR effluent.

## Graphical abstract

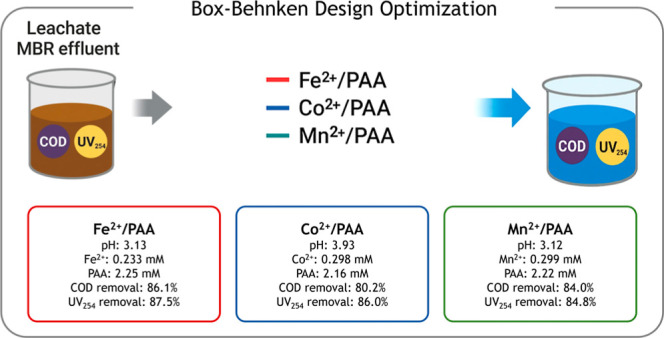

## 1 Introduction

In recent years, advanced oxidation processes (AOPs) have been widely used for the removal of resistant organic matter from wastewater.^1,2^ In AOPs, oxidants, known as peroxides (hydrogen peroxide (H_2_O_2_), peroxymonosulfate (PMS), and peroxydisulfate), form reactive species responsible for the oxidation of pollutants.^3^ For the generation of these reactive species showing high activity in the degradation of organic matter, peroxides have to be activated. Although various methods are used to activate peroxides, the addition of transition metals (Fe^2+^, Co^2+^, Cu^2+^, Mn^2+^, etc.) stands out among these methods due to its excellent catalytic activity and low energy requirement. In the reaction between peroxides and transition metals, an electron transfer occurs, and during the reactions, homolytic cleavage of the peroxide bond (O–O) leads to the formation of free radicals such as hydroxyl and sulfate radicals.^4^

In addition to peroxides, an oxidant, peracetic acid (CH_3_C­(O)­OOH, PAA), has started to be used in advanced wastewater treatment for the degradation of resistant organic matter.^5^ Commercial PAA solutions are commonly a mixture of PAA, H_2_O_2_, CH_3_C­(=O)­OH, and water.^5^ Despite the high redox potential (1.06–1.96 V) of PAA, it is not effective in the oxidation of resistant organic pollutants and requires activation as peroxides. UV application, transition metal addition, and heterocatalytic methods are used for PAA activation.^6–11^ Activation of PAA mainly results in the formation of acetyl­(per)­oxyl (CH_3_C­(O)­O^•^ and CH_3_C­(O)­OO^•^) and/or hydroxyl radicals (HO^•^).^9^ In PAA, the cleavage of the O–O bond is thermally favorable because its bond energy is 159 kJ/mol, which is considerably lower than the bond energy of the O–O bond in H_2_O_2_ and PMS.^7^ The types and amounts of free radical species generated as a result of the PAA activation depend on the activation method. UV activation results in the formation of HO^•^ and oxygen-centered radicals (R–O^•^), whereas oxygen-centered radicals are dominant radical species in transition metal activation.^5,7^ Since transition metals can easily break the O–O bond of PAA, their application as activators plays a significant role in the breakdown of resistant organic matter.^12^

Transition metals are extensively used as catalysts and can be categorized as homogeneous and heterogeneous, both operating on similar activation principles.^13^ Apart from energy activation mechanisms, transition metals can undergo electron gain and loss, leading to a cyclic activation process.^13^ The activation of PAA with transition-metal catalysts (metal/PAA) resembles the classical Fenton process.^14^ The reaction equations for transition-metal/PAA interactions are provided in Text S1.

Studies in the literature on the removal of organic pollutants through PAA oxidation predominantly use synthetic solutions.^7,11,14–20^ He et al.^21^ achieved 58% naproxen (NPX) removal after 10 min with the Mn^2+^/PAA process at 5 μM NPX concentration, 10 μM Mn^2+^ dose, 500 μM PAA dose, pH 7.2 ± 0.5, and 25 ± 1 °C process conditions, and increased this efficiency to 100% by adding 20 mM NaHCO_3_. Li et al.^22^ investigated the effect of protocatechuic acid (PCA) addition on sulfamethoxazole (SMX) removal with the Fe-catalyzed PAA process, and they achieved <5% SMX removal efficiency with the Fe­(III)/PAA process at 5 μM pollutant concentration, 100 μM Fe­(III) dose, 200 μM PAA dose, and pH 7 process conditions, while SMX removal was increased to 95.3% with 125 μM PCA addition. Wu et al.^23^ investigated NPX removal with the Cu­(II)-catalyzed PAA process and achieved nearly 100% NPX degradation within 10 min at 10 μM pollutant concentration, 10 μM Cu­(II) dose, 500 μM PAA dose, pH 9.8, and 22 °C conditions. He et al.^24^ investigated the effect of using waste manganese sand (WMS) in PAA activation on the removal of emerging contaminants and reported nearly 100% NPX degradation after a 30 min reaction time at 5 μM contaminant concentration, 1.50 mM PAA dose, 1.00 g/L WMS dose, pH 8, and 25 ± 1 °C reaction conditions. On the other hand, there are several studies in the literature related to the disinfection of water and wastewater using PAA oxidation.^25–30^ However, to the best of our knowledge, no studies are present on the removal of resistant organic matter from wastewater using PAA oxidation. Besides, there is no study in the literature that evaluates the performance of different catalyst applications for PAA activation in leachate treatment.

In this study, the removal of resistant organic pollutants from the leachate membrane bioreactor (MBR) effluent by transition-metal-catalyzed PAA oxidation was investigated. In the study, the Box–Behnken design (BBD) was used to evaluate the effects of process variables and their interactions with each other. The main aim of the experimental design used for process modeling is to examine the relationships among various parameter levels and the response as a dependent variable. The goal is to maximize the information obtained from the study while minimizing the number of experiments needed to reach the optimum conditions. This study is unique and innovative in terms of applying Fe^2+^/PAA, Co^2+^/PAA, and Mn^2+^/PAA processes to real wastewater and using BBD for the optimization of operating parameters.

## 2 Materials and Methods

### 2.1 Characterization of MBR Effluent Leachate and Analytical Methods

The wastewater treated in this study was obtained from the MBR effluent of the leachate treatment plant located at the Kömürcüoda Sanitary Landfill Facility in Istanbul, Türkiye. To maintain chemical stability and inhibit any undesirable biological or chemical reactions, the collected effluents were preserved at +4 °C. The characterization of the wastewater and the used methods are given in Table 1. MBR effluent analyses were carried out according to the standard methods recommended by APHA.^31^ The closed reflux titrimetric method was used for the determination of chemical oxygen demand (COD). WTW Multi 9620 IDS was used to measure the pH and conductivity. UV_254_ and color analyses were performed using a WTW Photolab 6600 UV/vis spectrophotometer. To prevent interference between PAA and H_2_O_2_ during COD analyses, all samples were pretreated with Na_2_S_2_O_5_ according to the ratios specified by Leite et al.^32^ before conducting the COD analysis.

**Table 1:** Characterization of MBR Effluent and Analytical Methods

Parameter	Value	Unit	Method
pH	8.56	-	WTW Multi 9620 IDS device
Conductivity	21.2	mS/cm	WTW Multi 9620 IDS device
COD	2143	mg/L	APHA 5220-C
Color	6631	Pt–Co, mg/L	WTW 6600 UV–vis spectrophotometer
UV_254_	23.9	1/cm	WTW 6600 UV–vis spectrophotometer

All chemicals used in the experimental studies were of analytical grade. PAA solution (38–40%, Merck) was used as an oxidant. Sodium hydroxide (NaOH, purity: >99%) and sulfuric acid (H_2_SO_4_, purity: 95–97%), which were used to adjust pH levels, were obtained from Merck. FeSO_4_.7H_2_O (purity: 99.5–104.5%, Sigma-Aldrich), Ni­(NO_3_)_2_.6H_2_O (purity >97%, Sigma-Aldrich), CoCl_2_.6H_2_O (purity >99.0–102%, Supelco), ZnSO_4_.7H_2_O (purity: 99.5–103%, Merck), MnSO_4_.H_2_O (purity >97%, Tekkim), and CuSO_4_.5H_2_O (purity: 99.0–100.5%, Merck) were used as catalysts. Na_2_S_2_O_5_ (purity >98.0–100.5%, Supelco) was used to remove excess PAA and H_2_O_2_ before COD analyses.

### 2.2 Experimental Studies

The experimental flowchart of the metal/PAA processes is shown in Figure 1. The experimental studies were conducted in a 500 mL beaker with 200 mL of wastewater samples. Before each experiment, the initial pH of the samples was adjusted by using 6 N H_2_SO_4_ or NaOH. Samples with an adjusted pH were placed into a jar test setup. After adding the oxidant and catalyst, the samples were rapidly mixed at 200 rpm for 1 min and then slowly mixed at 40 rpm for predetermined reaction times. Once the process was complete, the pH levels of the samples were adjusted to a predetermined level on the basis of the metal catalyst used. Finally, the samples were centrifuged at 4000 rpm for 5 min and the supernatant was separated. To remove PAA and H_2_O_2_, Na_2_S_2_O_5_ was added to the supernatant and mixed for 5 min according to the ratios specified by Leite et al.^32^ The supernatant was then separated, and the samples were prepared for the analysis. To determine the effect of transition metals on the PAA oxidation process, control experiments were conducted with six different transition metals. In control experiments, in which PAA was used as the oxidant and Fe^2+^, Zn^2+^, Cu^2+^, Mn^2+^, Ni^2+^, and Co^2+^ were used as catalysts, both single and combined processes were conducted.

**Figure 1:**
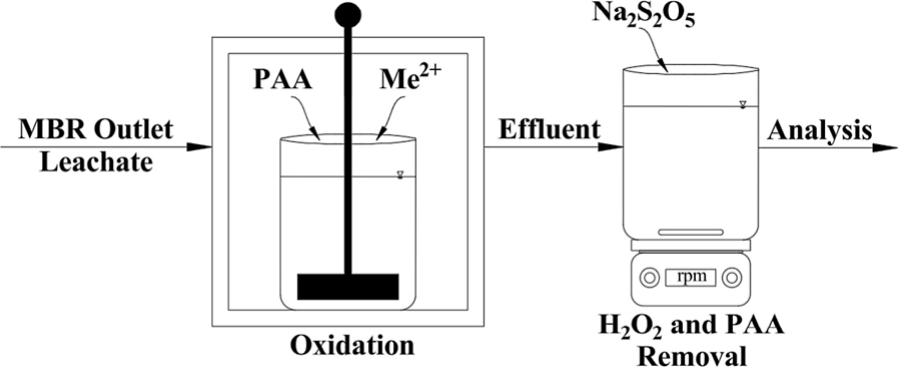
Experimental flowchart of Me^2+^/PAA processes.

### 2.3 Control Experiments

Control experiments were conducted for the alternative catalyst-activated PAA oxidation application to the leachate MBR effluent, which has a high content of resistant organic matter. The COD removal efficiencies obtained in the control experiments are shown in Figure 2. In the control experiments, the COD removal efficiency was investigated for 6 different metal catalysts (Fe^2+^, Zn^2+^, Cu^2+^, Mn^2+^, Ni^2+^, and Co^2+^) under the same conditions. For this purpose, experiments were conducted at pH 3, an oxidant dose of 1 mM, a catalyst dose of 0.1 mM, and a reaction time of 30 min. First, COD removal efficiency was evaluated using Fe^2+^, Zn^2+^, Cu^2+^, Mn^2+^, Ni^2+^, Co^2+^, and PAA alone. Then, Me^2+^/PAA processes were applied for each metal catalyst, and their performances were compared. The COD removal efficiencies determined by the application of single processes ranged between 7.5% and 21.2%, while the removal efficiencies of the combined processes ranged between 51.3% and 85.2%. Among the catalysts, Fe^2+^, Mn^2+^, and Co^2+^ were selected to achieve the highest COD removal efficiencies. Before modeling the process conditions of Fe^2+^/PAA, Mn^2+^/PAA, and Co^2+^/PAA processes, where the highest COD removal efficiency was obtained, experiments were carried out at varying pH values and reaction times to determine the model value ranges. Although not shown in the graphs separately, experiments were also conducted at different PAA concentrations. By application of these preliminary experiments, the model value ranges of the most effective processes were revealed.

**Figure 2:**
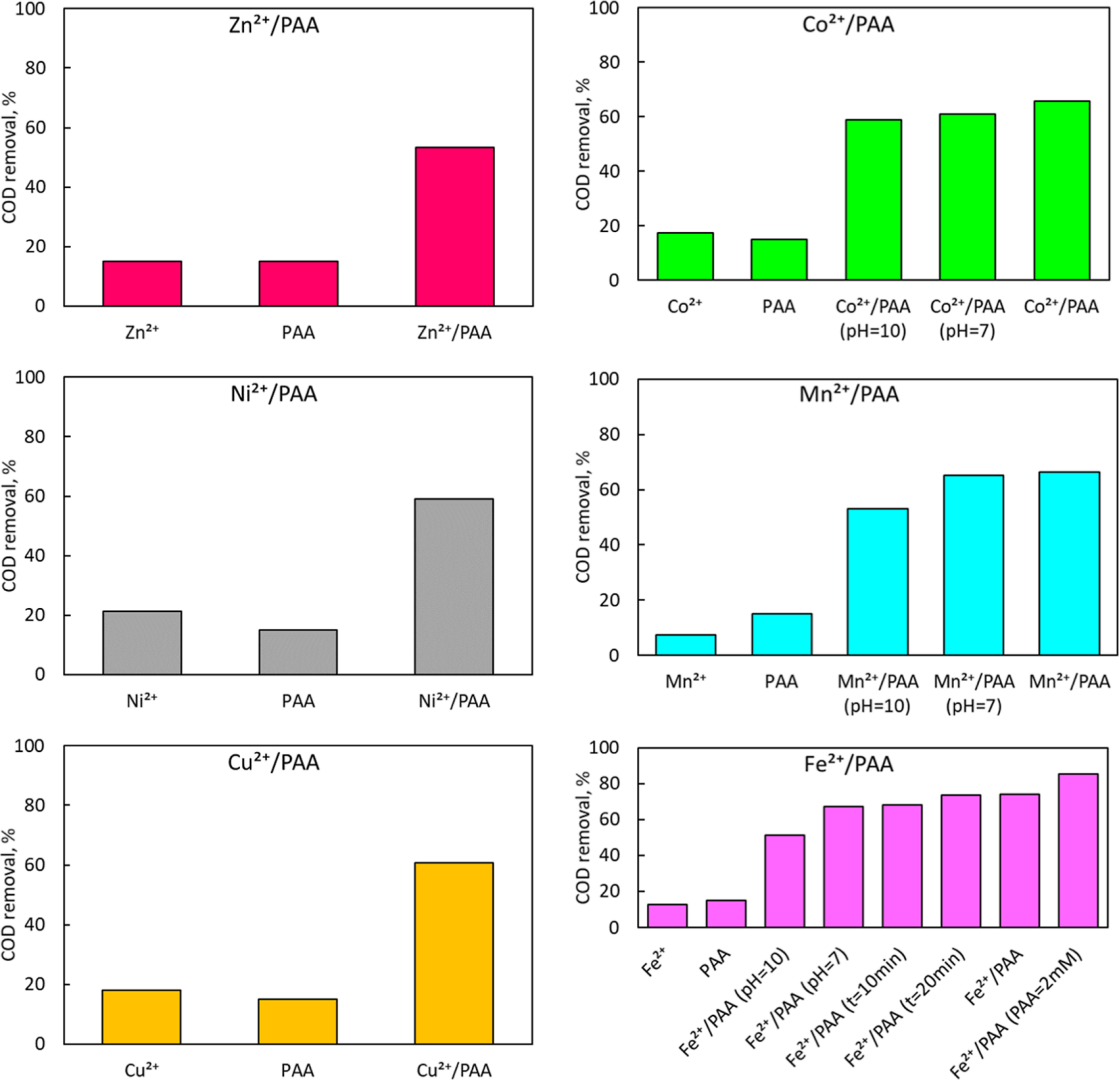
COD removal efficiency in the control experiments (conditions: pH = 3, oxidant dose = 1 mM, catalyst dose = 0.1 mM, reaction time = 30 min. If there is a different condition, they are shown in brackets).

The significantly higher catalytic activity of Fe^2+^, Co^2+^, and Mn^2+^ compared to Zn^2+^, Cu^2+^, and Ni^2+^ can be attributed to their higher capability to undergo continuous electron gain and loss, leading to a highly efficient cyclic activation process.^14^ The effective activation of PAA relies on the continuous cleavage of its O–O bond to generate reactive radicals.^5,9^ This cleavage is dependent on the easily reversible oxidation states of the catalyst, such as the Fe^2+^/Fe^3+^, Co^2+^/Co^3+^, and Mn^2+^/Mn^3+^ redox cycles.^14^ Unlike Fe, Co, and Mn, the other applied metals showed lower pollutant removal efficiencies under these specific operating conditions. For instance, it was reported that the direct activation of PAA by Cu^2+^ to form reactive species is typically very slow, and the thermodynamically limited transformation from Cu­(II) to Cu­(I) acts as a rate-limiting step.^23^ Similarly, Zn^2+^ and Ni^2+^ showed lower PAA activation in the applied system. This may be due to the lack of an efficient electron exchange between PAA and the metals, which is required to maintain the reactions.^14^ Consequently, this leads to lower free radical generation and the observed lower COD removal efficiencies for these metals.

### 2.4 Experimental Design and Optimization of Metal/PAA Processes

Following the control experiments, the operating conditions (oxidant dose, catalyst dose, and initial pH) for the Me^2+^/PAA (Me = Fe^2+^, Mn^2+^, and Co^2+^) processes, which showed the highest COD removal efficiency in control experiments, were optimized using a three-factor, three-level BBD. For each of the Fe^2+^/PAA, Mn^2+^/PAA, and Co^2+^/PAA processes, 15 experimental sets were conducted. The range of values for the variables, determined based on the data from the control experiments, was coded as −1, 0, and +1. The variables and their ranges are presented in Table 2.

**Table 2:** Variables and Their Ranges of the Me^2+^/PAA Processes

Factor	Original factor	–1	0	+1
Initial pH	A	3	6	9
Catalyst (Fe^2+^, Co^2+^, Mn^2+^) dose, mM	B	0.1	0.2	0.3
Oxidant (PAA) dose, mM	C	0.75	1.5	2.25

## 3 Results and Discussion

### 3.1 Model Development and Statistical Analysis

The relationship between system responses and the operational parameters (initial pH value, catalyst dose, and oxidant dose) for the Fe^2+^/PAA, Co^2+^/PAA, and Mn^2+^/PAA processes was modeled using the BBD. The experimental design matrix and the predicted and experimentally obtained COD and UV_254_ removal efficiencies for each set of operating conditions in the matrix are presented in Table 3. Based on experimental design results, regression equations with coded variables were obtained using Design Expert. The system responses, COD and UV_254_ removal efficiencies, were calculated by using the constant coefficients of three linear (A, B, C), three interactive (AB, AC, BC), and three quadratic (A^2^, B^2^, C^2^) parameters given in eqs 1–6.

1 $${COD}_{{Fe}^{2+}/PAA}removal,\%=+75.57-9.06A+5.50B+8.85C+1.73AB+1.53AC+0.0191BC-0.5677A^{2}-0.3994B^{2}-3.47C^{2}$$

2 $${{UV}_{254,}}_{{Fe}^{2+}/PAA}removal,\%=+84.85-6.40A+1.32B+8.17C+3.38AB+1.75AC+0.6065BC-4.25A^{2}-2.30B^{2}-2.02C^{2}$$

3 $${COD}_{{Co}^{2+}/PAA}removal,\%=+63.82-4.66A+3.40B+6.74C+0.5349AB-1.29AC+1.16BC+3.54A^{2}+4.42B^{2}-1.64C^{2}$$

4 $${{UV}_{254,}}_{{Co}^{2+}/PAA}removal,\%=+72.08-4.62A+3.19B+5.37C+2.25AB+0.8834AC+1.84BC+4.58A^{2}+4.29B^{2}-2.43C^{2}$$

5 $${COD}_{{Mn}^{2+}/PAA}removal,\%=+62.17-4.99A+4.80B+6.06C+0.9730AB+1.07AC-0.5604BC+0.9922A^{2}+6.97B^{2}+4.09C^{2}$$

6 $${{UV}_{254,}}_{{Mn}^{2+}/PAA}removal,\%=+74.66-4.73A+4.59B+5.63C+1.49AB+0.7109AC-0.7081BC-2.44A^{2}+3.29B^{2}+1.21C^{2}$$

**Table 3:** Model Matrix and Experimental-Predicted Model Results for the Processes

				Fe^2+^/PAA				Co^2+^/PAA				Mn^2+^/PAA			
Set	Factor			COD removal, %		UV_254_ removal, %		COD removal, %		UV_254_ removal, %		COD removal, %		UV_254_ removal, %	
	A	B	C	exp.	pred.	exp.	pred.	exp.	pred.	exp.	pred.	exp.	pred.	exp.	pred.
1	3	0.1	1.5	79.2	79.9	86.5	86.8	73.7	73.6	84.8	84.6	72.2	71.3	77.8	77.1
2	9	0.1	1.5	58.2	58.3	68.8	69.7	64.1	63.2	71.6	70.8	58.6	59.4	63.6	64.7
3	3	0.3	1.5	87.5	87.4	91.0	90.1	78.3	79.3	85.8	86.5	79.7	78.9	84.4	83.3
4	9	0.3	1.5	73.4	72.8	76.9	76.6	71.0	71.1	81.6	81.8	70.0	70.9	76.2	76.9
5	3	0.2	0.75	72.3	73.3	79.1	79.8	63.2	62.3	75.1	74.4	66.8	67.2	72.5	73.2
6	9	0.2	0.75	50.6	52.1	60.9	61.0	55.6	55.6	63.5	63.3	56.3	55.1	63.3	62.3
7	3	0.2	2.25	89.4	87.9	92.7	92.6	78.4	78.4	83.2	83.3	76.1	77.2	82.1	83.1
8	9	0.2	2.25	73.9	72.8	81.6	80.8	65.7	66.5	75.1	75.8	69.9	69.4	75.8	75.0
9	6	0.1	0.75	59.0	57.4	71.4	70.4	56.7	57.6	66.3	67.2	61.4	61.8	68.3	68.2
10	6	0.3	0.75	69.2	68.3	74.1	74.3	62.3	62.1	69.9	69.9	72.3	72.5	78.5	78.8
11	6	0.1	2.25	74.1	75.0	85.7	85.5	68.6	68.8	74.3	74.3	75.3	75.0	81.2	80.9
12	6	0.3	2.25	84.4	86.1	90.9	91.8	78.8	77.9	85.3	84.3	83.9	83.5	88.6	88.7
13	6	0.2	1.5	74.5	75.6	86.2	84.8	65.1	63.8	74.2	72.1	63.5	62.2	74.2	74.6
14	6	0.2	1.5	76.8	75.6	82.9	84.8	63.9	63.8	72.7	72.1	61.0	62.2	76.6	74.6
15	6	0.2	1.5	75.3	75.6	85.5	84.8	62.4	63.8	69.3	72.1	62.0	62.2	73.2	74.6

The most crucial aspect of data analysis is evaluating the model’s adequacy. To determine the model’s significance and suitability, Analysis of Variance (ANOVA) was used, and the results of ANOVA are presented in Table 4. The detailed results for each process are given in Tables S1–S3. Testing the model adequacy, coefficient of determination (*R* ^2^), adjusted *R* ^2^ (Adj. *R* ^2^), predicted *R* ^2^ (Pred. *R* ^2^) values, alongside coefficient of variation (CV), adequate precision (AP), and lack of fit values were evaluated. The *R* ^2^ values obtained for Fe^2+^/PAA, Co^2+^/PAA, and Mn^2+^/PAA processes are close to 1, and the Adj. *R* ^2^ values are also close to their respective *R* ^2^ values. These values, being close to 1 and each other, indicate a high correlation between the predicted and experimental data. *R* ^2^ values close to 1 suggest that most of the data variation can be explained by the model. Additionally, the difference between Pred. *R* ^2^ and Adj. *R* ^2^ values should ideally be less than 0.20 ^33^. For all three processes and both response variables, the difference between the Pred. *R* ^2^ and Adj. *R* ^2^ values were determined to be lower than 0.2. The model is statistically acceptable, as evidenced by the high *F*-values, *p*-values below 0.05, and lack of fit prob-*F* values above 0.05 ^34^. ANOVA results given in Table S1 show that pH and PAA dose linear variables are highly significant, and Fe^2+^ dose is significant for COD and UV_254_ removal in the Fe^2+^/PAA process. For the Co^2+^/PAA process, pH and Co^2+^ dose are significant, and the PAA dose is highly significant for COD removal, while all linear parameters are significant for UV_254_ removal (Table S2). It is seen from Table S3 that pH and Mn^2+^ dose linear parameters are significant and PAA dose is highly significant for COD removal, whereas all linear parameters are significant for UV_254_ removal in the Mn^2+^/PAA process.

**Table 4:** ANOVA Results for the Response Surface Model^a^

Parameter	Fe^2+^/PAA		Co^2+^/PAA		Mn^2+^/PAA	
	COD removal, %	UV_254_ removal %	COD removal, %	UV_254_ removal %	COD removal, %	UV_254_ removal %
SS	1591.39	1140.84	770.94	690.40	914.27	685.60
MS	176.82	126.76	85.66	76.71	101.59	76.18
*F* value	51.29	59.03	49.58	23.43	51.32	30.87
*P* value	0.0002	0.0002	0.0002	0.0014	0.0002	0.0007
*R* ^2^	0.9893	0.9907	0.9889	0.9768	0.9893	0.9823
adj. *R* ^2^	0.9700	0.9739	0.9690	0.9351	0.9700	0.9505
pred. *R* ^2^	0.8519	0.9219	0.8886	0.8696	0.8778	0.8384
AP	23.6239	26.4442	22.0742	15.6818	24.7347	20.5146
CV, %	2.54	1.81	1.96	2.40	2.05	2.07

a SS: sum of squares, MS: mean square, AP: adequate precision, and CV: coefficient of variation.

One of the parameters used to assess model adequacy, the AP value, being greater than 4, indicates a high ability of the model to predict the pollutant removal efficiency within the design space. For all of the models, AP values were found to be greater than 4. Another indicator of model reliability, the CV value, which signifies repeatability, is ideally less than 10%. The CV values obtained for the models of the Fe^2+^/PAA, Co^2+^/PAA, and Mn^2+^/PAA processes were found to be less than 10 (Table 4).

The accuracy of the data can be assessed by using parity and normal probability plots. As presented in Figures S1–S6, the residual values show a distribution around a straight line. This indicates that the developed models fit the experimental results of Fe^2+^/PAA, Co^2+^/PAA, and Mn^2+^/PAA processes. In Figures S1–S6, predicted and experimentally obtained data for COD and UV_254_ removal by Fe^2+^/PAA, Co^2+^/PAA, and Mn^2+^/PAA processes were also provided. The agreement between predicted and experimental removal efficiencies, as shown by data clustering around the 45° line in the predicted versus actual value plots, demonstrates high consistency between model predictions and experimental results. All these findings indicate that BBD is a reliable and applicable tool for optimizing Fe^2+^/PAA, Co^2+^/PAA, and Mn^2+^/PAA processes applied for COD and UV_254_ removal from leachate MBR effluent.

### 3.2 Effect of Independent Variables

To show the effects of independent variables on system responses within the selected ranges and their interactions, 3-D graphs of the regression models were drawn. When interactions between independent variables are significant, the curvature of the three-dimensional surface becomes distinctly apparent. The plots show prominent peak values, indicating optimum conditions that yield maximum pollutant removal efficiencies, dependent on all variables.^35^ These peak values provide a maximum pollutant removal efficiency. Deviating from these points results in decreased removal efficiencies; therefore, excess or deficiency of these values for independent variables is undesirable. Figures 3, 4 and 5 show the effects of operational parameters on Fe^2+^/PAA, Co^2+^/PAA, and Mn^2+^/PAA processes from the 3-D graphs. In these graphs, one independent variable is held constant, while the effects of the other two variables within specified ranges on system responses are observed

**Figure 3:**
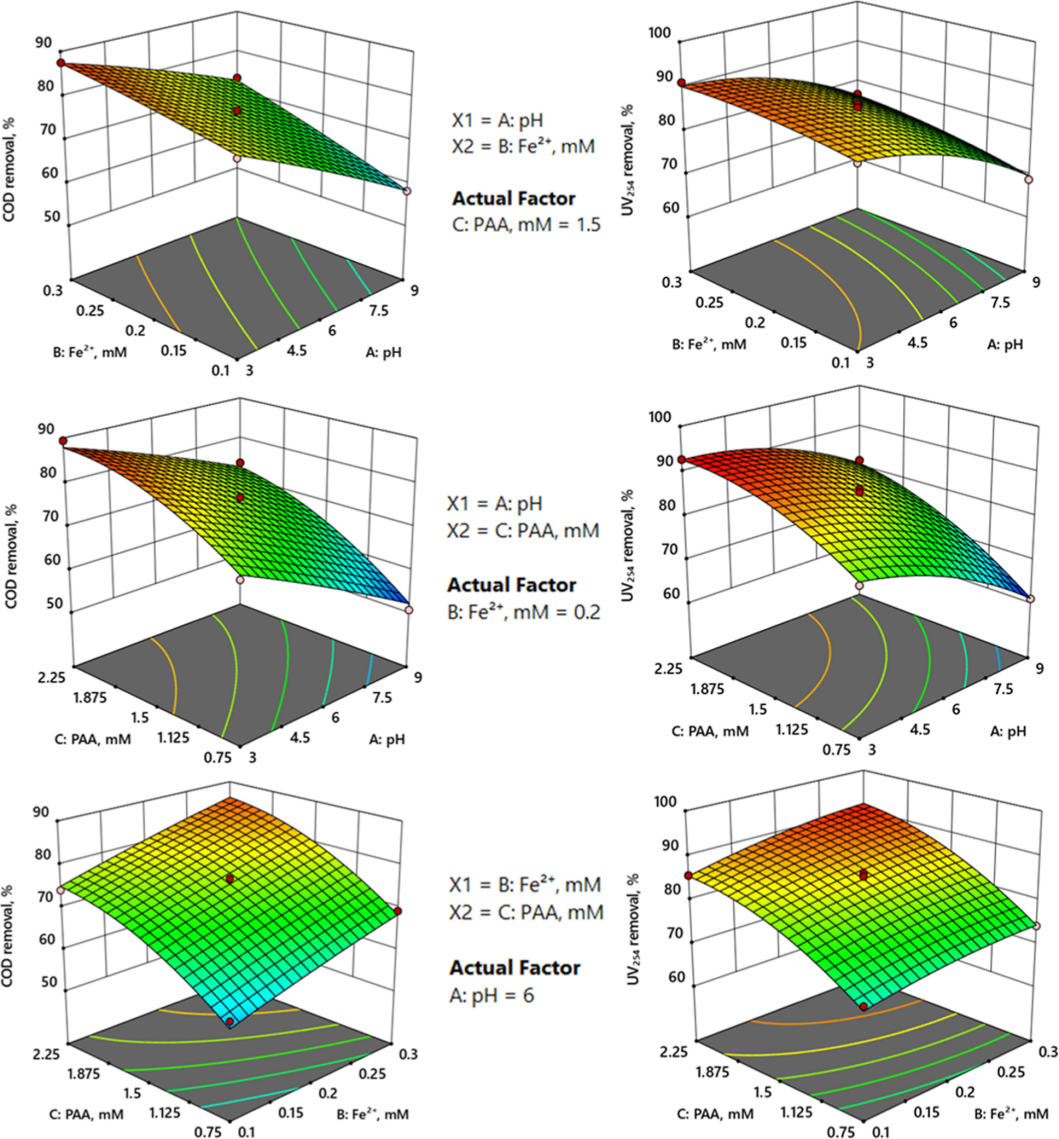
Response surface plots for the Fe^2+^/PAA process.

**Figure 4:**
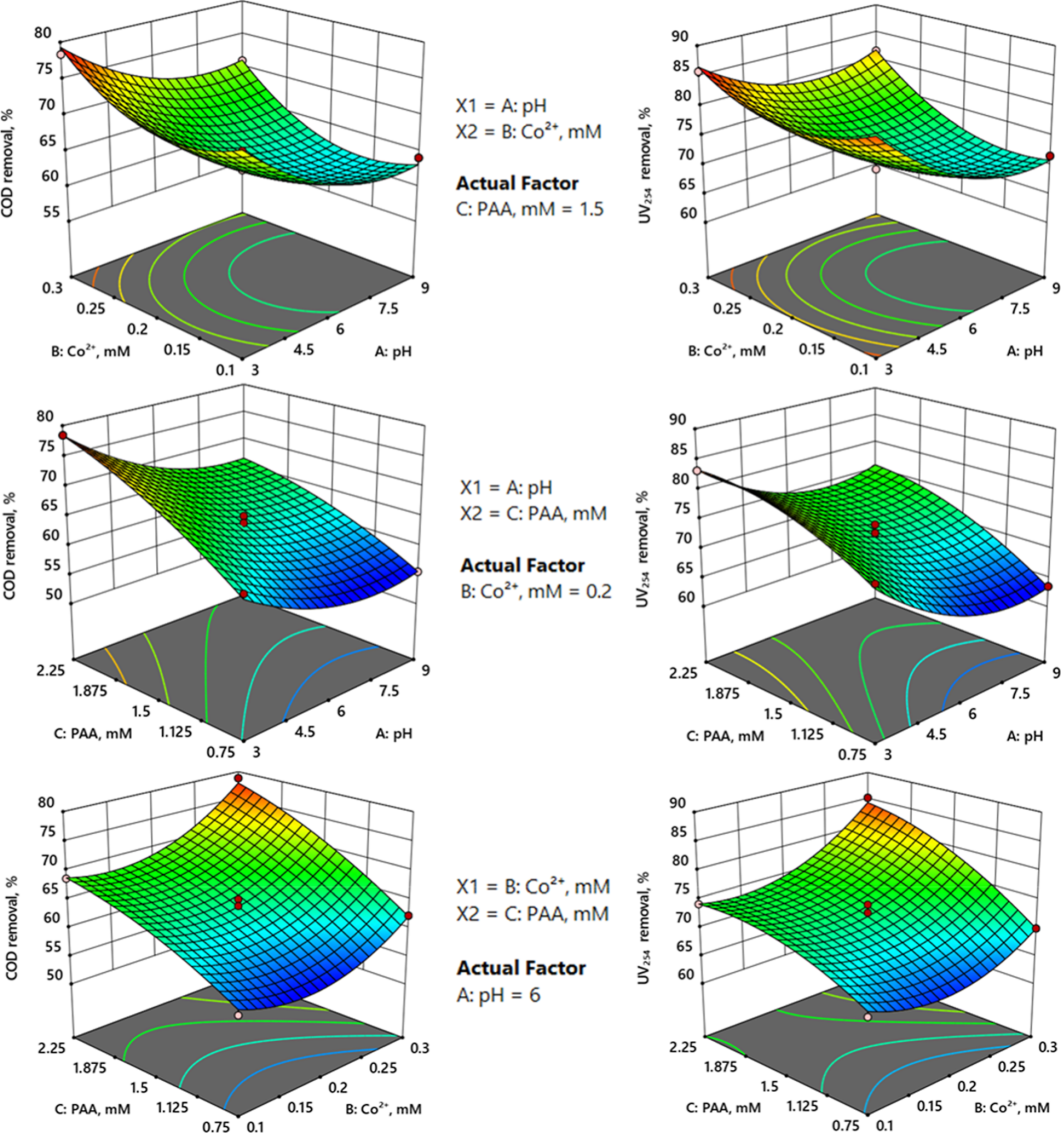
Response surface plots for the Co^2+^/PAA process.

**Figure 5:**
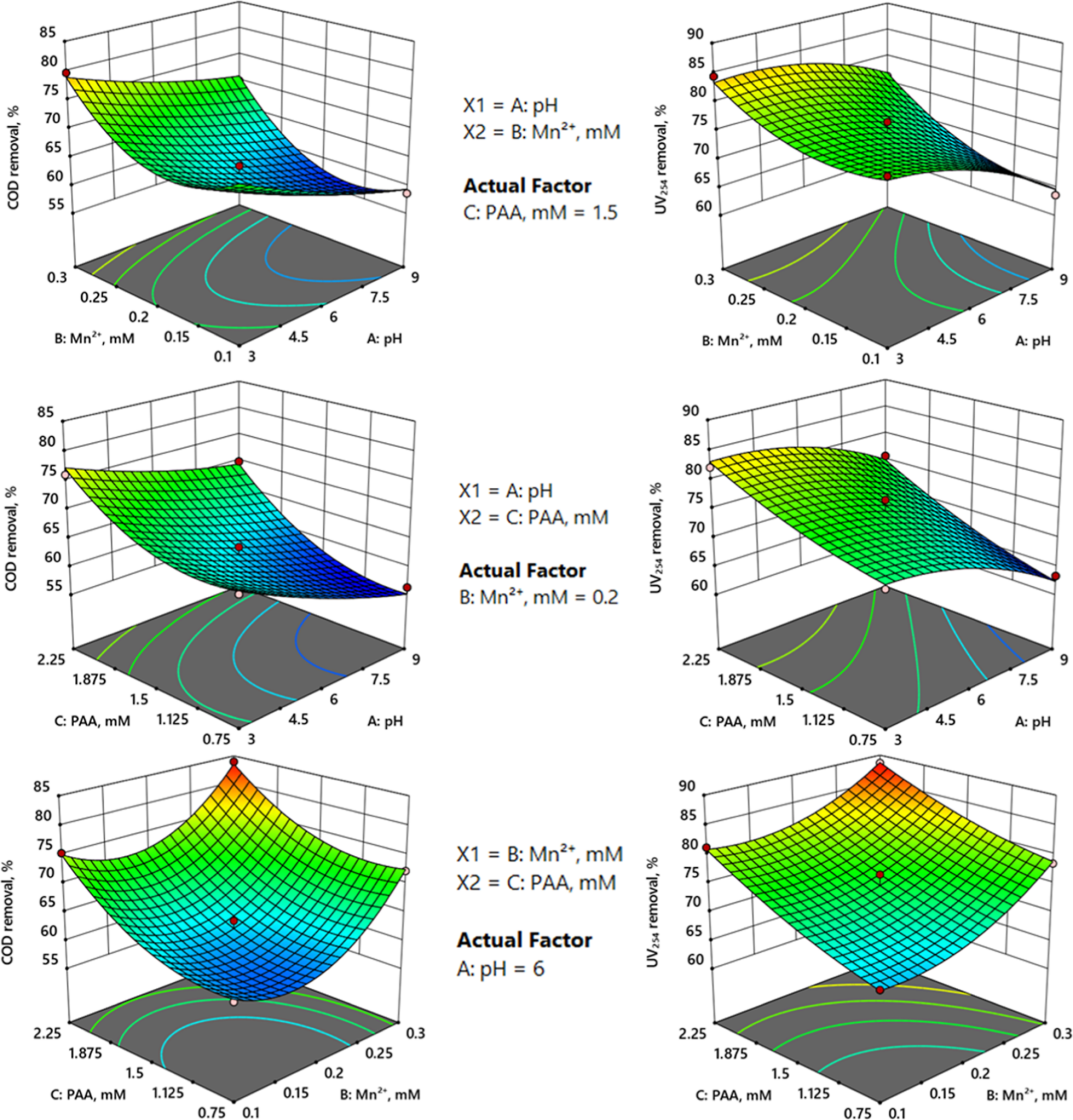
Response surface plots for the Mn^2+^/PAA process.

The initial pH value, one of the independent variables, positively affects pollutant removal efficiencies up to an optimum value. However, beyond this optimum point, a slight decrease in removal efficiency is observed. In all AOPs, there is a direct relationship between the removal of organic pollutants and the pH of the medium. The pH of the solution or wastewater is also crucial for the activation of PAA by transition metals. As detailed in the reactions provided in Text S1, the activation of PAA by transition metals involves the generation of OH^–^ and H^+^ ions (Text S1, eqs 1 and 2).^14^ Specifically, in the catalytic cycles of Co, Mn, and Fe, the formation of radical species is accompanied by the release of OH^–^ (Text S1, eqs 3, 7, and 15) and H^+^ (Text S1, eqs 4, 8, and 18).^4,8,36^ Therefore, an excess of H^+^ ions at very low pH or an excess of OH^–^ ions at high pH can suppress these forward reactions, thereby limiting free radical generation. Furthermore, as noted for the Fe^2+^/PAA system, the rate of hydroxyl radical formation through the Fe^2+^/PAA reaction is much higher in the pH range of 3–7 compared to the Fe^2+^/H_2_O_2_ process, making PAA the dominant source of reactive radicals.^8^ The initial pH of the wastewater or solution determines the solubility of the metal ions. At low pH values, metal ions are soluble, but at higher pH values, they tend to form hydroxyl complexes or precipitate as hydroxides. The form of the metal ions directly affects the activation of PAA. Furthermore, the form of PAA varies based on the pH value of the solution. In acidic and slightly alkaline conditions, PAA exists predominantly in its neutral form (PAA°), whereas in alkaline conditions, the stable form is the PAA¯ molecule.^7–9^ The neutral form of PAA shows better oxidation potential compared to its anionic form.^37^ At high pH values, PAA can self-decompose, and its concentration can decrease due to the consumption of H_2_O_2_.^12^ Transition metal activation in PAA oxidation is effective in the pH range of 3–7. Optimum pH ranges of 3–7 have also been determined for the Fe^2+^/PAA, Co^2+^/PAA, and Mn^2+^/PAA processes.

Another significant parameter in the transition-metal activation of PAA is the oxidant dose. As seen from Figures 3–5, pollutant removal efficiencies increase with increasing PAA dose. Higher concentrations of PAA result in more free radical formation. More organic matter reacts with more free radicals, leading to degradation and a reduction in pollution concentrations. However, beyond the optimum oxidant dose, a quenching effect is observed, which causes a decrease in radical concentration. The PAA solution contains H_2_O_2_; hence, increasing the PAA dose also increases the H_2_O_2_ dose. Increasing the PAA dose not only enhances free radical formation due to PAA activation but also increases the concentration of H_2_O_2_, which can consume free radicals. However, it should be noted that at higher PAA doses, the rate of free radical formation due to PAA activation is generally higher than the rate at which radicals are consumed by H_2_O_2_.^8,12^ For all three processes, the optimum PAA dose was found near the maximum value within the specified range (Figures 3–5). In this study, a quenching effect was not observed because the PAA dose did not exceed the optimum value.

Another significant parameter affecting pollutant removal in these processes is the catalyst dose.^5^ As the catalyst dose increases, pollutant removal efficiencies, PAA decomposition rates, and reaction rates all increase.^12^ This is because higher catalyst doses activate more PAA, leading to increased free radical formation and, consequently, enhancing pollutant degradation. However, this increase is not indefinite. Beyond the optimum catalyst dose, a decrease in pollutant removal efficiencies is observed (Figures 3–5). This decrease can be explained by the reaction of excess metal ions with free radicals. In the Fe^2+^/PAA system, excessive Fe^2+^ ions react with hydroxyl radicals, reducing the concentration of hydroxyl radicals.^12^ Kim et al.^9^ reported that the inhibition of carbamazepine degradation when the PAA dose was lower than the cobalt dose might be due to the improvement of radical scavenging by extra cobalt ions.

The standardized effects of independent variables on the dependent variables and their interactions with each other in the Fe^2+^/PAA, Co^2+^/PAA, and Mn^2+^/PAA processes were determined by using Pareto plots (Figure 6). In the Pareto plot, the length of each bar indicates the magnitude of the standardized effect of each factor on the response.^38^ Parameters with shorter bars are interpreted as having lower contributions to the removal efficiency. As presented in Figure 6, all linear parameters significantly affect COD and UV_254_ removal in Fe^2+^/PAA, Co^2+^/PAA, and Mn^2+^/PAA processes, with the highest impact being the pH value for COD removal and PAA dose for UV_254_ removal by the Fe^2+^/PAA process; with the highest impact being the PAA dose for COD removal and UV_254_ removal by the Co^2+^/PAA process; and with the highest impact being the PAA dose for COD removal and UV_254_ removal by the Co^2+^/PAA and Mn^2+^/PAA processes.

**Figure 6:**
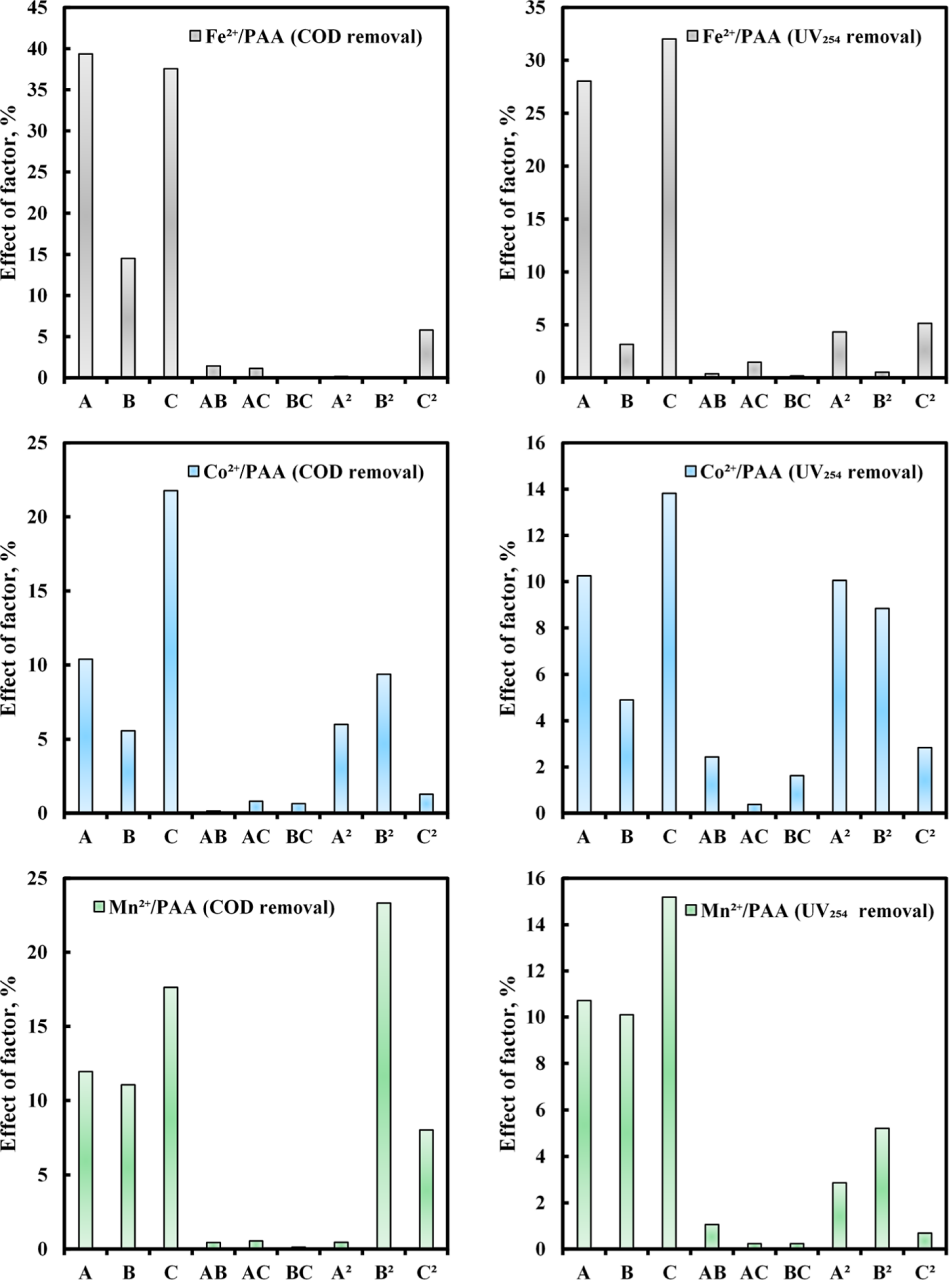
Pareto charts of COD and UV_254_ removal by Me^2+^/PAA processes (A is the initial pH, B is the catalyst dose, and C is the oxidant dose. A, B, and C are linear terms; AB, AC, and BC are interactive terms; A^2^, B^2^, and C^2^ are quadratic terms).

The contribution of linear, interactive, and quadratic terms was also validated by the contribution rate graphs presented in Figure 7. In the contribution rate graphs, each term’s sum of squares value is divided by the total sum of squares to show the contribution rate of the parameters. The data indicate that linear terms have the highest contribution percentages, while interactive terms contribute the least. This implies that interactions between independent variables have a minimal effect on predicting pollutant removal efficiencies. In evaluating the total contribution percentages of quadratic, linear, and interactive terms, linear parameters are the most significant. Although further detailed studies are needed, the current findings suggest a direct and linear relationship between parameters and COD and UV_254_ removals using the Fe^2+^/PAA, Co^2+^/PAA, and Mn^2+^/PAA processes. The low contribution of interactive terms indicates that combined behaviors of variables do not dominate the processes and suggests that complex reactions between variables do not lead to processes. Model equation coefficients, Pareto analysis, and ANOVA results were found to be consistent with each other.

**Figure 7:**
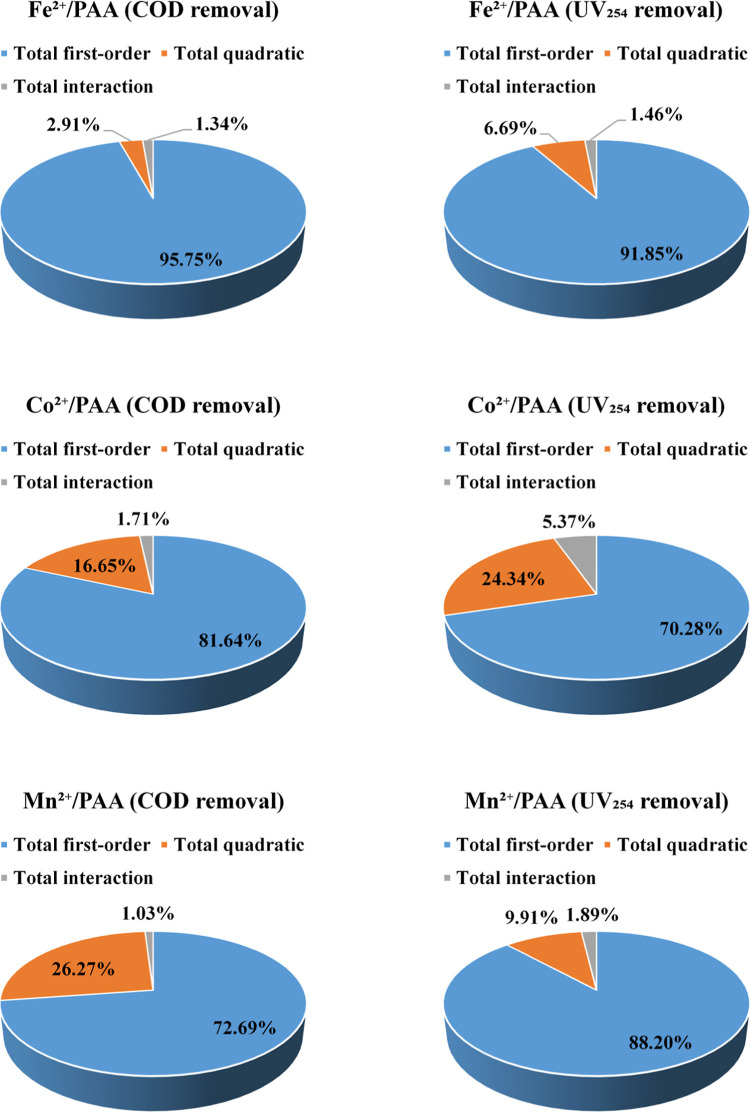
Contribution percentages of linear, interactive, and quadratic terms in COD and UV_254_ removal by Me^2+^/PAA processes.

### 3.3 Model Optimization

To achieve maximum pollutant removal efficiencies, it is important to determine the optimum operating conditions for each process. Numerical analysis and the Design Expert software were used to identify the optimum values within the experimental range for achieving maximum pollutant removal efficiencies. Under the optimum conditions for the Fe^2+^/PAA process (pH: 3.13, Fe^2+^ dose: 0.233 mM, and PAA dose: 2.25 mM), COD and UV_254_ removal efficiencies were predicted as 88.9% and 90.9%, respectively, by the model. Experimental results confirmed these predictions with actual COD and UV_254_ removal efficiencies of 86.1% and 87.5%, respectively. BBD-based models for the Fe^2+^/PAA process were quite close to the actual experimental results (2.8% error for COD removal and 3.4% error for UV_254_ removal). For the Co^2+^/PAA process under optimum conditions (pH: 3.93, Co^2+^ dose: 0.298 mM, and PAA dose: 2.16 mM), the model predicted COD and UV_254_ removal efficiencies of 82.4% and 87.1%, respectively, whereas experimental results under these conditions showed COD and UV_254_ removal efficiencies of 80.2% and 86.0%, respectively. BBD-based models for the Co^2+^/PAA process were quite close to the actual experimental results (2.2% error for COD removal and 1.1% error for UV_254_ removal). For the Mn^2+^/PAA process under optimum conditions (pH: 3.12, Mn^2+^ dose: 0.299 mM, and PAA dose: 2.22 mM), the model predicted COD and UV_254_ removal efficiencies of 86.8% and 88.6%, respectively, and experimental results for COD and UV_254_ removal efficiencies were 84.0% and 84.8%, respectively. BBD-based models for the Mn^2+^/PAA process were quite close to the actual experimental results (2.8% error for COD removal and 3.8% error for UV_254_ removal). The close agreement between predicted and experimental data under optimum conditions shows that the BBD approach effectively determines the optimum process conditions for achieving the highest removal efficiency. For the Fe^2+^/PAA process, the standard deviation (STD) values for the COD and UV_254_ removal efficiencies were 1.86 and 1.46, respectively, while the standard error (SE) values were 1.42 and 1.12, respectively. For the Co^2+^/PAA process, the STD values for the COD and UV_254_ removal efficiencies were 1.31 and 1.81, respectively, and the SE values were 0.76 and 1.04, respectively. For the Mn^2+^/PAA process, the STD values for the COD and UV_254_ removal efficiencies were 1.41 and 1.57, respectively, while the SE values were 0.81 and 0.91, respectively. These results indicate that the model demonstrates consistent predictability within a 95% confidence interval (*p* < 0.05), showing low STD and SE values.

### 3.4 Process Mechanisms and Comparison with the Literature

PAA is attracting increasing attention due to the necessity of reducing chlorine usage in disinfection processes and controlling disinfection byproducts. In addition, PAA is of interest due to its high oxidation potential, especially in the treatment of refractory pollutants such as pharmaceutical active ingredients.^5^ Thus, PAA has recently started to be used in AOPs with various activation methods. With the activation of PAA, in addition to hydroxyl radicals, acetyl­(per)­oxyl radicals are also formed and contribute to the degradation of organic compounds.^9^ The mechanism of this process is driven by the cyclic electron transfer of transition metals, as detailed in the chemical equations provided in Text S1. The general interaction between transition metals and PAA leads to the cleavage of the O–O bond, forming CH_3_C­(O)­O^•^ and CH_3_C­(O)­OO^•^radicals (Text S1, eqs 1 and 2).^14^ Specifically, in the activation by Co^2+^ (Text S1, eqs 3–6) and Mn^2+^ (Text S1, eqs 7–14), the generated organic radicals can further react to form methyl radicals.^4,36^ The Fe^2+^-catalyzed mechanism is more complex, which concurrently generates HO^•^ and high-valent iron species (Text S1, eqs 15–28).^8^ Furthermore, H_2_O_2_, which is in equilibrium with PAA, also reacts with Fe^2+^ to form additional HO^•^(Text S1, eqs 29–32).^8^

Metal catalysts are effectively and widely used as activators in AOPs. Among these, iron-based catalysts are mostly preferred due to their low cost, high efficiency, and environmental friendliness.^39^ The effectiveness of Co-based catalysts in PAA activation has been shown in many studies in organic matter removal from synthetic solutions.^7,9^ Radical and nonradical pathway pollutant removals have been reported with Mn-based PAA oxidation.^21,40^ While previous studies in the literature have primarily focused on the removal of specific micropollutants from synthetic solutions or natural water matrices, such as the highly efficient degradation of naproxen and sulfamethoxazole using Mn, Fe, or Cu activated PAA processes,^8,12,20–23^ the present study demonstrated the effectiveness of these processes on a complex real wastewater matrix. The optimized Fe^2+^/PAA, Mn^2+^/PAA, and Co^2+^/PAA processes in this study achieved high COD removal efficiencies of 86.1%, 84.0%, and 80.2%, respectively, in biologically treated leachate. These findings indicate that the strong catalytic performances of Fe, Co, and Mn reported in the literature for specific pollutants were also effective in the treatment of recalcitrant organic matter in real wastewater. Recent comprehensive reviews have highlighted that the practical application, interactions with complex matrices, and scalability of PAA-based AOPs in real wastewaters remain a knowledge gap that requires investigation.^5,14^ Therefore, the successful application of these processes to real wastewater in the present study shows a potential and fills a crucial gap in the literature.

## 4 Conclusion

In this study, the performance of combined AOPs (Fe^2+^/PAA, Co^2+^/PAA, and Mn^2+^/PAA) in the removal of COD and UV_254_ from leachate MBR effluent was investigated. In the initial step of the study, single and binary processes were applied to evaluate the effect of different catalysts on PAA activation. Fe^2+^/PAA, Ni^2+^/PAA, Zn^2+^/PAA, Co^2+^/PAA, Cu^2+^/PAA, and Mn^2+^/PAA processes were compared in terms of COD removal efficiencies, and Fe^2+^/PAA, Co^2+^/PAA, and Mn^2+^/PAA processes, which showed the highest COD removal efficiencies, were optimized. BBD was employed to optimize independent variables, including initial pH, catalyst dose, and oxidant dose, while the system responses were COD and UV_254_ removal efficiencies. Under the optimum conditions for the Fe^2+^/PAA process (pH: 3.13, Fe^2+^ dose: 0.233 mM, and PAA dose: 2.25 mM), the COD and UV_254_ removal efficiencies were predicted by the model as 88.9% and 90.9%, respectively. The experimental results validated these predictions with 86.1% and 87.5% efficiency, respectively. For the Co^2+^/PAA process under optimum conditions (pH: 3.93, Co^2+^ dose: 0.298 mM, and PAA dose: 2.16 mM), the model predicted COD and UV_254_ removal efficiencies as 82.4% and 87.1%, respectively, whereas the experimental results showed efficiencies of 80.2% and 86.0%, respectively. Under the optimum conditions for the Mn^2+^/PAA process (pH: 3.12, Mn^2+^ dose: 0.299 mM, and PAA dose: 2.22 mM), the model predicted COD and UV_254_ removal efficiencies as 86.8% and 88.6%, respectively, and the experimental results yielded efficiencies of 84.0% and 84.8%, respectively. The final COD concentration (298 mg/L for the Fe^2+^/PAA process, 424 mg/L for the Co^2+^/PAA process, and 343 mg/L for the Mn^2+^/PAA process) meets the receiving environment discharge standard (COD <500 mg/L) according to the water pollution control regulation of Türkiye. It was concluded that Fe^2+^/PAA, Co^2+^/PAA, and Mn^2+^/PAA processes are effective for COD and UV_254_ removal from leachate MBR effluent, and the models developed using BBD for these three processes have high statistical significance.

## Supplementary Material



## Notes

Additional text for transition metal/PAA reactions; tables for ANOVA results of the Fe^2+^/PAA, Co^2+^/PAA, and Mn^2+^/PAA processes; and parity and normal distribution plots for COD and UV_254_ removal by the Fe^2+^/PAA, Co^2+^/PAA, and Mn^2+^/PAA processes (PDF)

## References

[1] Lee J., Von Gunten U., Kim J.-H. (2020). Persulfate-Based Advanced Oxidation:
Critical Assessment of Opportunities and Roadblocks. Environ. Sci. Technol..

[2] Li Y., Dong H., Li L., Tang L., Tian R., Li R., Chen J., Xie Q., Jin Z., Xiao J. (2021). Recent Advances in Waste Water Treatment through Transition Metal
Sulfides-Based Advanced Oxidation Processes. Water Res..

[3] Oturan M. A., Aaron J.-J. (2014). Advanced Oxidation
Processes in Water/Wastewater Treatment:
Principles and Applications. A Review. Crit.
Rev. Environ. Sci. Technol..

[4] Zhao Z., Li X., Li H., Qian J., Pan B. (2021). New Insights into the
Activation of Peracetic Acid by Co (II): Role of Co (II)-Peracetic
Acid Complex as the Dominant Intermediate Oxidant. ACS ES&T Eng..

[5] Ao X., Eloranta J., Huang C.-H., Santoro D., Sun W., Lu Z., Li C. (2021). Peracetic Acid-Based Advanced Oxidation Processes for
Decontamination and Disinfection of Water: A Review. Water Res..

[6] Cai M., Sun P., Zhang L., Huang C.-H. (2017). UV/Peracetic Acid for Degradation
of Pharmaceuticals and Reactive Species Evaluation. Environ. Sci. Technol..

[7] Wang Z., Wang J., Xiong B., Bai F., Wang S., Wan Y., Zhang L., Xie P., Wiesner M. R. (2020). Application of Cobalt/Peracetic
Acid to Degrade Sulfamethoxazole at Neutral Condition: Efficiency
and Mechanisms. Environ. Sci. Technol..

[8] Kim J., Zhang T., Liu W., Du P., Dobson J. T., Huang C.-H. (2019). Advanced Oxidation Process with Peracetic
Acid and
Fe (II) for Contaminant Degradation. Environ.
Sci. Technol..

[9] Kim J., Du P., Liu W., Luo C., Zhao H., Huang C.-H. (2020). Cobalt/Peracetic
Acid: Advanced Oxidation of Aromatic Organic Compounds by Acetylperoxyl
Radicals. Environ. Sci. Technol..

[10] Zhou F., Lu C., Yao Y., Sun L., Gong F., Li D., Pei K., Lu W., Chen W. (2015). Activated Carbon Fibers as an Effective
Metal-Free Catalyst for Peracetic Acid Activation: Implications for
the Removal of Organic Pollutants. Chem. Eng.
J..

[11] Wu W., Tian D., Liu T., Chen J., Huang T., Zhou X., Zhang Y. (2020). Degradation
of Organic Compounds
by Peracetic Acid Activated with Co3O4: A Novel Advanced Oxidation
Process and Organic Radical Contribution. Chem.
Eng. J..

[12] Wang S., Wang H., Liu Y., Fu Y. (2020). Effective Degradation
of Sulfamethoxazole with Fe2+-Zeolite/Peracetic Acid. Sep. Purif. Technol..

[13] Shi C., Li C., Wang Y., Guo J., Barry S., Zhang Y., Marmier N. (2022). Review of Advanced
Oxidation Processes Based on Peracetic
Acid for Organic Pollutants. Water.

[14] Correa-Sanchez S., Peñuela G. A. (2022). Peracetic Acid-Based Advanced Oxidation Processes for
the Degradation of Emerging Pollutants: A Critical Review. J. Water Proc. Eng..

[15] Ike I. A., Linden K. G., Orbell J. D., Duke M. (2018). Critical Review of
the Science and Sustainability of Persulphate Advanced Oxidation Processes. Chem. Eng. J..

[16] García-Cervilla R., Santos A., Romero A., Lorenzo D. (2020). Remediation of Soil
Contaminated by Lindane Wastes Using Alkaline Activated Persulfate:
Kinetic Model. Chem. Eng. J..

[17] Zhi D., Lin Y., Jiang L., Zhou Y., Huang A., Yang J., Luo L. (2020). Remediation of Persistent Organic Pollutants in Aqueous Systems by
Electrochemical Activation of Persulfates: A Review. J. Environ. Manage..

[18] Zhou X., Wu H., Zhang L., Liang B., Sun X., Chen J. (2020). Activation
of Peracetic Acid with Lanthanum Cobaltite Perovskite for Sulfamethoxazole
Degradation under a Neutral PH: The Contribution of Organic Radicals. Molecules.

[19] Zhang L., Fu Y., Wang Z., Zhou G., Zhou R., Liu Y. (2021). Removal of
Diclofenac in Water Using Peracetic Acid Activated by Zero Valent
Copper. Sep. Purif. Technol..

[20] Zhou R., Zhou G., Liu Y., Liu S., Wang S., Fu Y. (2022). Activated Peracetic Acid by Mn3O4 for Sulfamethoxazole Degradation:
A Novel Heterogeneous Advanced Oxidation Process. Chemosphere.

[21] He L., Zou J., Wu J., Li S., Wu Z., Huang Y., Kou X., Cheng Q., Wang P., Ma J. (2024). Highly Efficient Degradation
of Emerging Contaminants with Sodium Bicarbonate-Enhanced Mn (II)/Peracetic
Acid Process: Formation and Contribution of Mn (V). Environ. Sci. Technol..

[22] Li S., Zou J., Wu J., Lin J., Tang C., Yang S., Chen L., Li Q., Wang P., Ma J. (2024). Protocatechuic
Acid Enhanced the Selective Degradation of Sulfonamide Antibiotics
in Fe (III)/Peracetic Acid Process under Actually Neutral PH Conditions. Water Res..

[23] Wu J., Zou J., Lin J., Li S., He L., Wu Z., Li Q., Gong C., Ma J. (2024). Overlooked Role of Coexistent Hydrogen
Peroxide in Activated Peracetic Acid by Cu (II) for Enhanced Oxidation
of Organic Contaminants. Environ. Sci. Technol..

[24] He L., Zou J., Wu Z., Li S., Li J., Wu J., Yang Z., Li Q., Ma J. (2025). Waste Manganese Sand
Activated Peracetic Acid for Efficient Degradation of Emerging Contaminants:
Resource Utilization and Dual-Phase Mechanism. Chem. Eng. J..

[25] Beber
de Souza J., Queiroz Valdez F., Jeranoski R. F., Vidal C. M. d. S., Cavallini G. S. (2015). Water and Wastewater Disinfection
with Peracetic Acid and UV Radiation and Using Advanced Oxidative
Process PAA/UV. Int. J. Photoenergy.

[26] Weng S., Dunkin N., Schwab K. J., McQuarrie J., Bell K., Jacangelo J. G. (2018). Infectivity
Reduction Efficacy of
UV Irradiation and Peracetic Acid-UV Combined Treatment on MS2 Bacteriophage
and Murine Norovirus in Secondary Wastewater Effluent. J. Environ. Manage..

[27] Hassaballah A. H., Bhatt T., Nyitrai J., Dai N., Sassoubre L. (2020). Inactivation
of E. Coli, Enterococcus Spp., Somatic Coliphage, and Cryptosporidium
Parvum in Wastewater by Peracetic Acid (PAA), Sodium Hypochlorite,
and Combined PAA-Ultraviolet Disinfection. Environ.
Sci..

[28] Hassaballah A. H., Nyitrai J., Hart C. H., Dai N., Sassoubre L. M. (2019). A Pilot-Scale
Study of Peracetic Acid and Ultraviolet Light for Wastewater Disinfection. Environ. Sci..

[29] Rizzo L., Agovino T., Nahim-Granados S., Castro-Alférez M., Fernández-Ibáñez P., Polo-López M. I. (2019). Tertiary
Treatment of Urban Wastewater by Solar and UV-C Driven Advanced Oxidation
with Peracetic Acid: Effect on Contaminants of Emerging Concern and
Antibiotic Resistance. Water Res..

[30] Zhang K., San Y., Cao C., Zhang T., Cen C., Zhou X. (2020). Optimising
the Measurement of Peracetic Acid to Assess Its Degradation during
Drinking Water Disinfection. Environ. Sci. Pollut.
Res..

[31] APHA Standard Methods for Examination of Water and Wastewater; American Water Works Association (AWWA, WEF and APHA), 2017.

[32] Leite L. de S., Tango M. D., Filho J. A. Z., Hoffmann M. T., Daniel L. A. (2021). Implications
of COD Analysis Use in the Peracetic Acid-Based Wastewater Treatment. Water Sci. Technol..

[33] Ahmadi M., Motlagh H. R., Jaafarzadeh N., Mostoufi A., Saeedi R., Barzegar G., Jorfi S. (2017). Enhanced Photocatalytic Degradation
of Tetracycline and Real Pharmaceutical Wastewater Using MWCNT/TiO2
Nano-Composite. J. Environ. Manage..

[34] Bajpai S., Gupta S. K., Dey A., Jha M. K., Bajpai V., Joshi S., Gupta A. (2012). Application
of Central Composite
Design Approach for Removal of Chromium (VI) from Aqueous Solution
Using Weakly Anionic Resin: Modeling, Optimization, and Study of Interactive
Variables. J. Hazard. Mater..

[35] Mohajeri S., Aziz H. A., Isa M. H., Zahed M. A., Adlan M. N. (2010). Statistical
Optimization of Process Parameters for Landfill Leachate Treatment
Using Electro-Fenton Technique. J. Hazard. Mater..

[36] Popov, E. ; Eloranta, J. ; Hietapelto, V. ; Vuorenpalo, V.-M. ; Aksela, R. ; Jäkärä, J. Mechanism of Decomposition of Peracetic Acid by Manganese Ions and Diethylenetriaminepentaacetic Acid (DTPA); Walter de Gruyte, 2005.

[37] Kiejza D., Kotowska U., Polińska W., Karpińska J. (2021). Peracids-New
Oxidants in Advanced Oxidation Processes: The Use of Peracetic Acid,
Peroxymonosulfate, and Persulfate Salts in the Removal of Organic
Micropollutants of Emerging Concern– A Review. Sci. Total Environ..

[38] Yetilmezsoy K., Demirel S., Vanderbei R. J. (2009). Response
Surface Modeling of Pb (II)
Removal from Aqueous Solution by Pistacia Vera L.: Box–Behnken
Experimental Design. J. Hazard. Mater..

[39] Baruah M. J., Dutta R., Zaki M. E. A., Bania K. K. (2024). Heterogeneous Iron-Based
Catalysts for Organic Transformation Reactions: A Brief Overview. Molecules.

[40] Rokhina E. V., Makarova K., Lahtinen M., Golovina E. A., Van As H., Virkutyte J. (2013). Ultrasound-Assisted
MnO2 Catalyzed Homolysis of Peracetic
Acid for Phenol Degradation: The Assessment of Process Chemistry and
Kinetics. Chem. Eng. J..

