# Supplementary material for: Modeling and Optimization of Transition Metal-Catalyzed Peracetic Acid Oxidation for Advanced Polishing of Biologically Treated Leachate
Source: ACS Omega. 2026 Jun 8;11(24):36194–206. doi: 10.1021/acsomega.6c03464 (PMC13295037; doi:10.1021/acsomega.6c03464)
Supplement: Supplementary file 1 [file ao6c03464_si_001.pdf]

Supporting Information

Modeling and Optimization of Transition Metal-Catalyzed Peracetic Acid Oxidation for Advanced Polishing of Biologically Treated Leachate

Emre Ünal[1], Ceyda Tunçak[1], Rabia Nefise Yılmaz[1], Emine Can-Güven[1*], Senem Yazici Guvenc[1], Gamze Varank[1]

[1] Yildiz Technical University, Faculty of Civil Engineering, Department of Environmental Engineering, 34220, Istanbul, Türkiye

Text S1

The reaction equations for transition metal/PAA interactions are provided in Equations 1-2 [1].

$$M^{n+} + CH_3C(O)OOH \rightarrow M^{(n+1)+} + CH_3C(O)O^{\bullet} + OH^{-} \quad (1)$$

$$M^{(n+1)+} + CH_3C(O)OOH \rightarrow M^{n+} + CH_3C(O)OO^{\bullet} + H^{+} \quad (2)$$

The available transition metal catalysts for PAA are predominantly Co, Fe, and Mn. The reactions occurring for the activation of PAA with Co^{2+} are given in Equations 3-6 [2].

$$Co^{2+} + CH_3C(O)OOH \rightarrow Co^{3+} + CH_3C(O)O^{\bullet} + OH^{-} \quad (3)$$

$$Co^{3+} + CH_3C(O)OOH \leftrightarrow Co^{2+} + CH_3C(O)OO^{\bullet} + H^{+} \quad (4)$$

$$CH_3C(O)O^{\bullet} \rightarrow CH_3^{\bullet} + CO_2 \quad (5)$$

$$CH_3^{\bullet} + O_2 \rightarrow CH_3O_2^{\bullet} \quad (6)$$

In the case of using Mn^{2+} as a catalyst to break the O-O bonds of PAA, the reactions given in Equations 7-14 occur [3].

$$Mn^{2+} + CH_3COOOH \rightarrow Mn^{3+} + CH_3COO^{\bullet} + OH^{-} \quad (7)$$

$$Mn^{3+} + CH_3COOOH \rightarrow Mn^{2+} + CH_3COOO^{\bullet} + H^{+} \quad (8)$$

$$CH_3COO^{\bullet} + CH_3COOOH \rightarrow CH_3COOH + CH_3COOO^{\bullet} \quad (9)$$

$$CH_3COOO^{\bullet} + CH_3COOOH \rightarrow CH_3COOH + CH_3COO^{\bullet} + O_2 \quad (10)$$

$$2CH_3COOO^{\bullet} \rightarrow 2CO_2 + O_2 + {}^{\bullet}CH_3 \quad (11)$$

$${}^{\bullet}CH_3 + O_2 \rightarrow CH_3OO^{\bullet} \quad (12)$$

$$CH_3OO^{\bullet} + CH_3COO^{\bullet} \rightarrow CH_2O + O_2 + CH_3COOH \quad (13)$$

$$2CH_3OO^{\bullet} \rightarrow O_2 + CH_2O + CH_3OH \quad (14)$$

The reactions in studies where Fe^{2+} is used as a catalyst for PAA activation are given in Equations 15-28 [4].

$$CH_3COOOH + Fe^{2+} \rightarrow CH_3COO^{\bullet} + Fe^{3+} + OH^{-} \quad (15)$$

$$CH_3COOOH + Fe^{2+} \rightarrow CH_3COO^- + Fe^{3+} + HO^\bullet \quad (16)$$

$$CH_3COOOH + Fe^{2+} \rightarrow CH_3COOH + Fe^{IV}O^{2+} \quad (17)$$

$$CH_3COOOH + Fe^{3+} \rightarrow CH_3COOO^\bullet + Fe^{2+} + H^+ \quad (18)$$

$$CH_3COOOH + CH_3COO^\bullet \rightarrow CH_3COOO^\bullet + CH_3COOH \quad (19)$$

$$H_2O_2 + CH_3COO^\bullet \rightarrow HO_2^\bullet + CH_3COOH \quad (20)$$

$$CH_3COOOH + HO^\bullet \rightarrow CH_3COOO^\bullet + H_2O \quad (21)$$

$$CH_3COOOH + HO^\bullet \rightarrow CH_3CO^\bullet + H_2O + O_2 \quad (22)$$

$$CH_3COOOH + HO^\bullet \rightarrow CH_3COOH + HO_2^\bullet \quad (23)$$

$$H_2O_2 + HO^\bullet \rightarrow HO_2^\bullet + H_2O \quad (24)$$

$$CH_3COOOH + 2Fe^{IV}O^{2+} \rightarrow CH_3COO^- + 2Fe^{3+} + OH^- + O_2 \quad (25)$$

$$Fe^{IV}O^{2+} + H_2O \rightarrow HO^\bullet + Fe^{3+} + OH^- \quad (26)$$

$$CH_3COO^\bullet \rightarrow {}^\bullet CH_3 + CO_2 \quad (27)$$

$$^\bullet CH_3 + O_2 \rightarrow CH_3OO^\bullet \quad (28)$$

In equilibrium with PAA, H_2O_2 also reacts with Fe^{2+} [4]. The reactions for H_2O_2/Fe^{2+} are given in Equations 29-32. In the pH range of 3-7, the rate of hydroxyl radical formation through the PAA/Fe^{2+} reaction is much higher than the reaction rate of the Fe^{2+}/H_2O_2 process, making PAA the dominant source of hydroxyl radicals [5].

$$H_2O_2 + Fe^{2+} \rightarrow HO^\bullet + Fe^{3+} + OH^- \quad (29)$$

$$H_2O_2 + Fe^{3+} \rightarrow HO_2^\bullet + Fe^{2+} + H^+ \quad (30)$$

$$H_2O_2 + Fe^{2+} \rightarrow H_2O + Fe^{IV}O^{2+} \quad (31)$$

$$H_2O_2 + Fe^{IV}O^{2+} \rightarrow HO_2^\bullet + Fe^{3+} + OH^- \quad (32)$$

Table S1. ANOVA results for the Fe^{2+}/PAA process

COD removal, %	SS	Df	MS	F value	P-value	Remark
A-pH	656.44	1	656.44	190.41	<0.0001	HS
B-Fe^{2+}, mM	242.26	1	242.26	70.27	0.0004	S
C-PAA, mM	626.41	1	626.41	181.70	<0.0001	HS
AB	11.99	1	11.99	3.48	0.1212	NS
AC	9.42	1	9.42	2.73	0.1592	NS
BC	0.0015	1	0.0015	0.0004	0.9844	NS
A^2	1.19	1	1.19	0.3451	0.5824	NS
B^2	0.5890	1	0.5890	0.1708	0.6965	NS
C^2	44.50	1	44.50	12.91	0.0157	S
Residual	17.24	5	3.45			
Lack of Fit	14.51	3	4.84	3.54	0.2279	NS
Pure Error	2.73	2	1.37			
Cor Total	1608.62	14				
UV_{254} removal, %	SS	Df	MS	F value	P-value	Remark
A-pH	467.94	1	467.94	217.90	<0.0001	HS
B-Fe^{2+}, mM	52.83	1	52.83	24.60	0.0042	S
C-PAA, mM	534.33	1	534.33	248.81	<0.0001	HS
AB	3.11	1	3.11	1.45	0.2829	NS
AC	12.21	1	12.21	5.69	0.0628	NS
BC	1.47	1	1.47	0.6851	0.4455	NS
A^2	33.31	1	33.31	15.51	0.0110	S
B^2	4.07	1	4.07	1.90	0.2271	NS
C^2	39.47	1	39.47	18.38	0.0078	S
Residual	10.74	5	2.15			
Lack of Fit	4.78	3	1.59	0.5352	0.7028	NS
Pure Error	5.96	2	2.98			
Cor Total	1151.58	14				

HS: Highly Significant, S: Significant, NS: Not Significant

Table S2. ANOVA results for the Co^{2+}/PAA process

COD removal, %	SS	Df	MS	F value	P-value	Remark
A-pH	173.49	1	173.49	100.43	0.0002	S
B-Co^{2+}, mM	92.73	1	92.73	53.67	0.0007	S
C-PAA, mM	363.15	1	363.15	210.20	<0.0001	HS
AB	1.14	1	1.14	0.6623	0.4527	NS
AC	6.69	1	6.69	3.87	0.1062	NS
BC	5.34	1	5.34	3.09	0.1391	NS
A^2	46.20	1	46.20	26.74	0.0036	S
B^2	72.23	1	72.23	41.81	0.0013	S
C^2	9.92	1	9.92	5.74	0.0619	NS
Residual	8.64	5	1.73			
Lack of Fit	4.90	3	1.63	0.8754	0.5723	NS
Pure Error	3.73	2	1.87			
Cor Total	779.57	14				

UV_{254} removal, %	SS	Df	MS	F value	P-value	Remark
A-pH	171.08	1	171.08	52.25	0.0008	S
B-Co^{2+}, mM	81.65	1	81.65	24.94	0.0041	S
C-PAA, mM	230.48	1	230.48	70.39	0.0004	S
AB	20.28	1	20.28	6.19	0.0552	S
AC	3.12	1	3.12	0.9533	0.3737	NS
BC	13.53	1	13.53	4.13	0.0978	NS
A^2	77.44	1	77.44	23.65	0.0046	S
B^2	68.06	1	68.06	20.79	0.0061	S
C^2	21.86	1	21.86	6.68	0.0492	S
Residual	16.37	5	3.27			
Lack of Fit	4.02	3	1.34	0.2173	0.8781	NS
Pure Error	12.35	2	6.17			
Cor Total	706.77	14				

HS: Highly Significant, S: Significant, NS: Not Significant

Table S3. ANOVA results for the Mn^{2+}/PAA process

COD removal, %	SS	Df	MS	F value	P-value	Remark
A-pH	199.29	1	199.29	100.68	0.0002	S
B-Mn^{2+}, mM	184.46	1	184.46	93.18	0.0002	S
C-PAA, mM	294.20	1	294.20	148.62	<0.0001	HS
AB	3.79	1	3.79	1.91	0.2252	NS
AC	4.60	1	4.60	2.32	0.1881	NS
BC	1.26	1	1.26	0.6347	0.4618	NS
A^2	3.64	1	3.64	1.84	0.2334	NS
B^2	179.57	1	179.57	90.72	0.0002	S
C^2	61.83	1	61.83	31.24	0.0025	S
Residual	9.90	5	1.98			
Lack of Fit	6.59	3	2.20	1.33	0.4565	NS
Pure Error	3.31	2	1.65			
Cor Total	924.17	14				
UV_{254} removal, %	SS	Df	MS	F value	P-value	Remark
A-pH	178.81	1	178.81	72.46	0.0004	S
B-Mn^{2+}, mM	168.59	1	168.59	68.32	0.0004	S
C-PAA, mM	253.34	1	253.34	102.66	0.0002	S
AB	8.87	1	8.87	3.60	0.1164	NS
AC	2.02	1	2.02	0.8191	0.4069	NS
BC	2.01	1	2.01	0.8126	0.4087	NS
A^2	22.03	1	22.03	8.93	0.0305	S
B^2	40.07	1	40.07	16.24	0.0100	S
C^2	5.37	1	5.37	2.17	0.2003	NS
Residual	12.34	5	2.47			
Lack of Fit	6.18	3	2.06	0.6693	0.6454	NS
Pure Error	6.16	2	3.08			
Cor Total	697.94	14				

HS: Highly Significant, S: Significant, NS: Not Significant

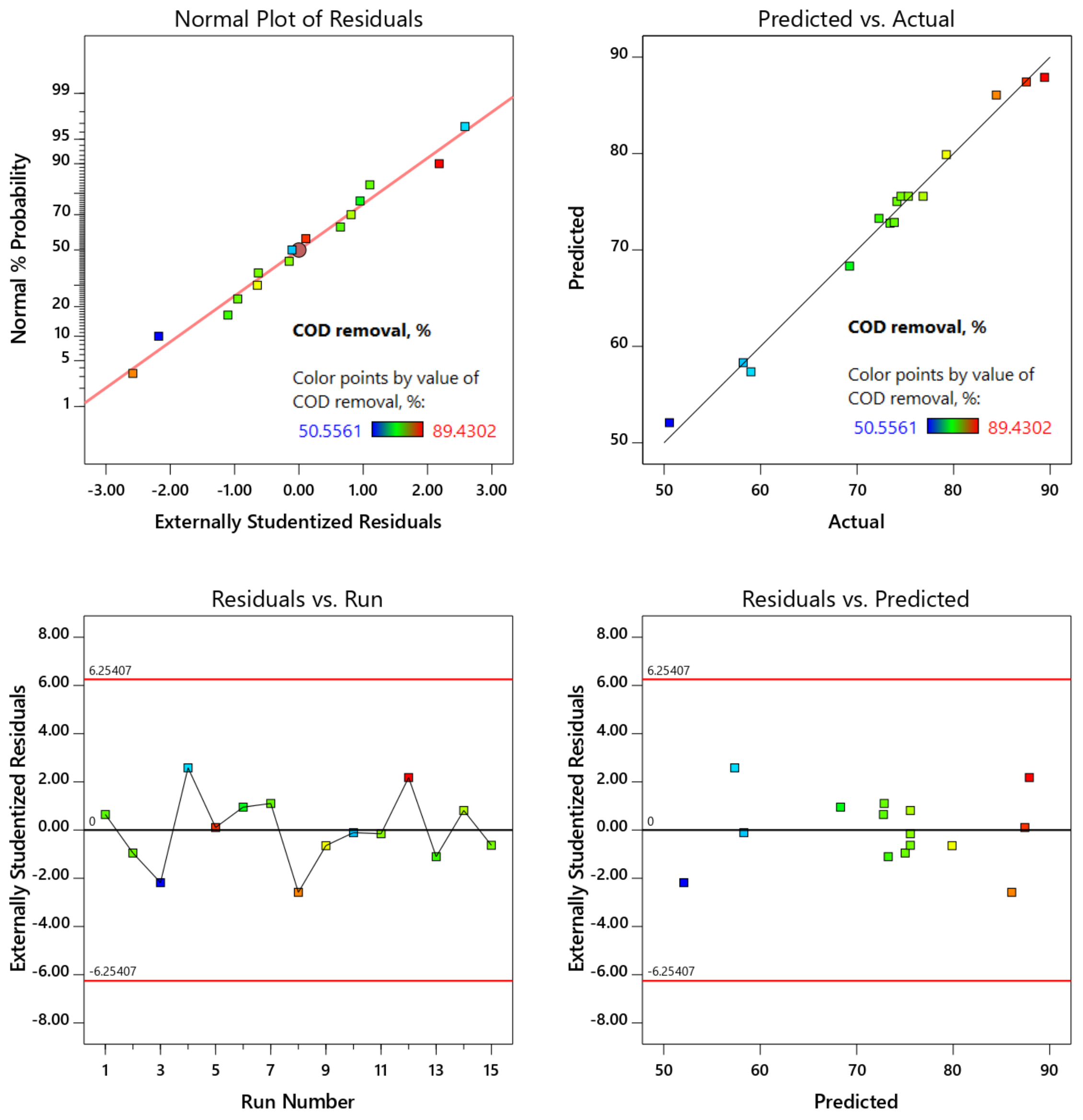

Figure S1. Parity and normal distribution plots for COD removal by the Fe^{2+}/PAA process

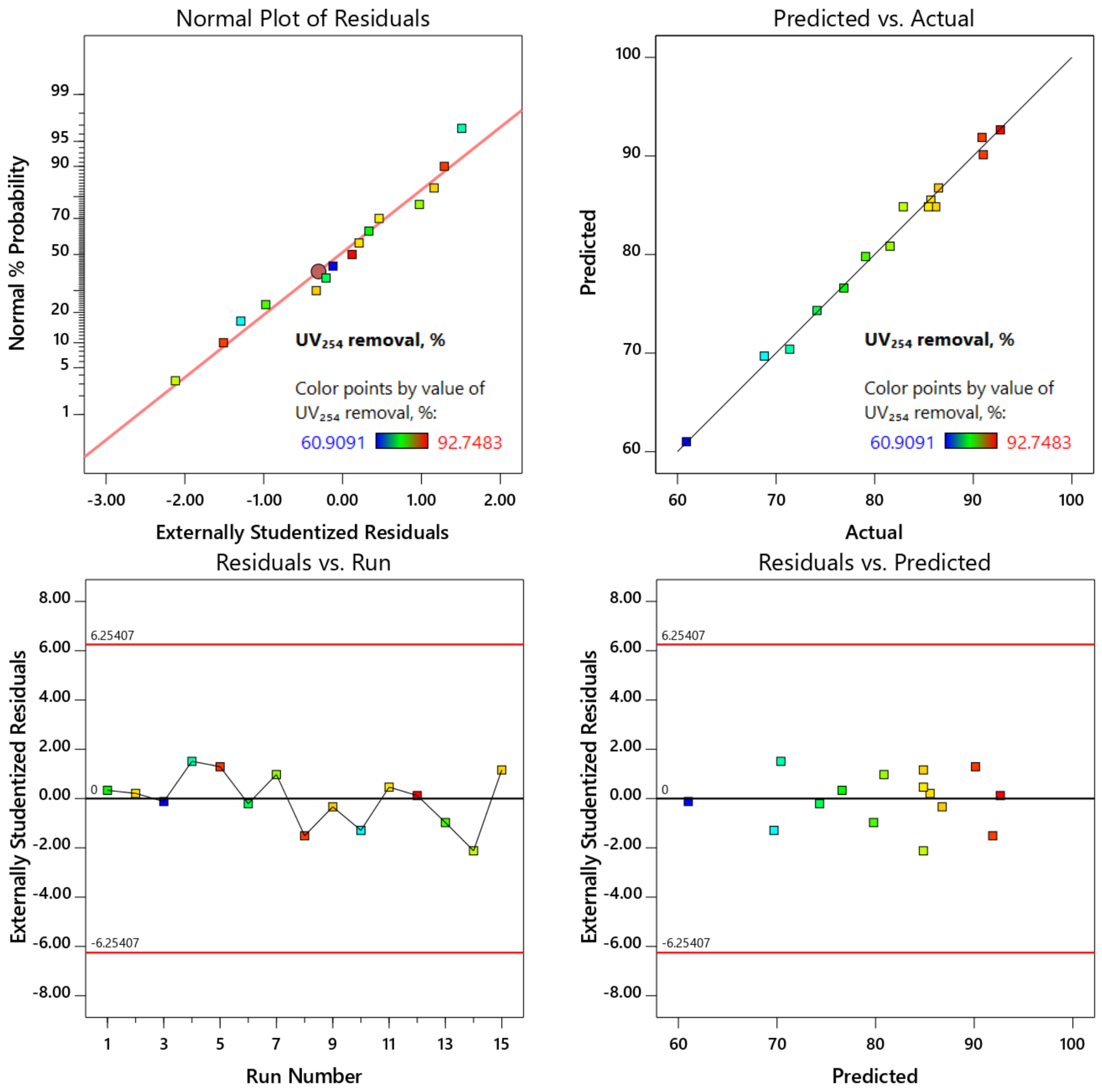

Figure S2. Parity and normal distribution plots for UV_{254} removal by the Fe^{2+}/PAA process

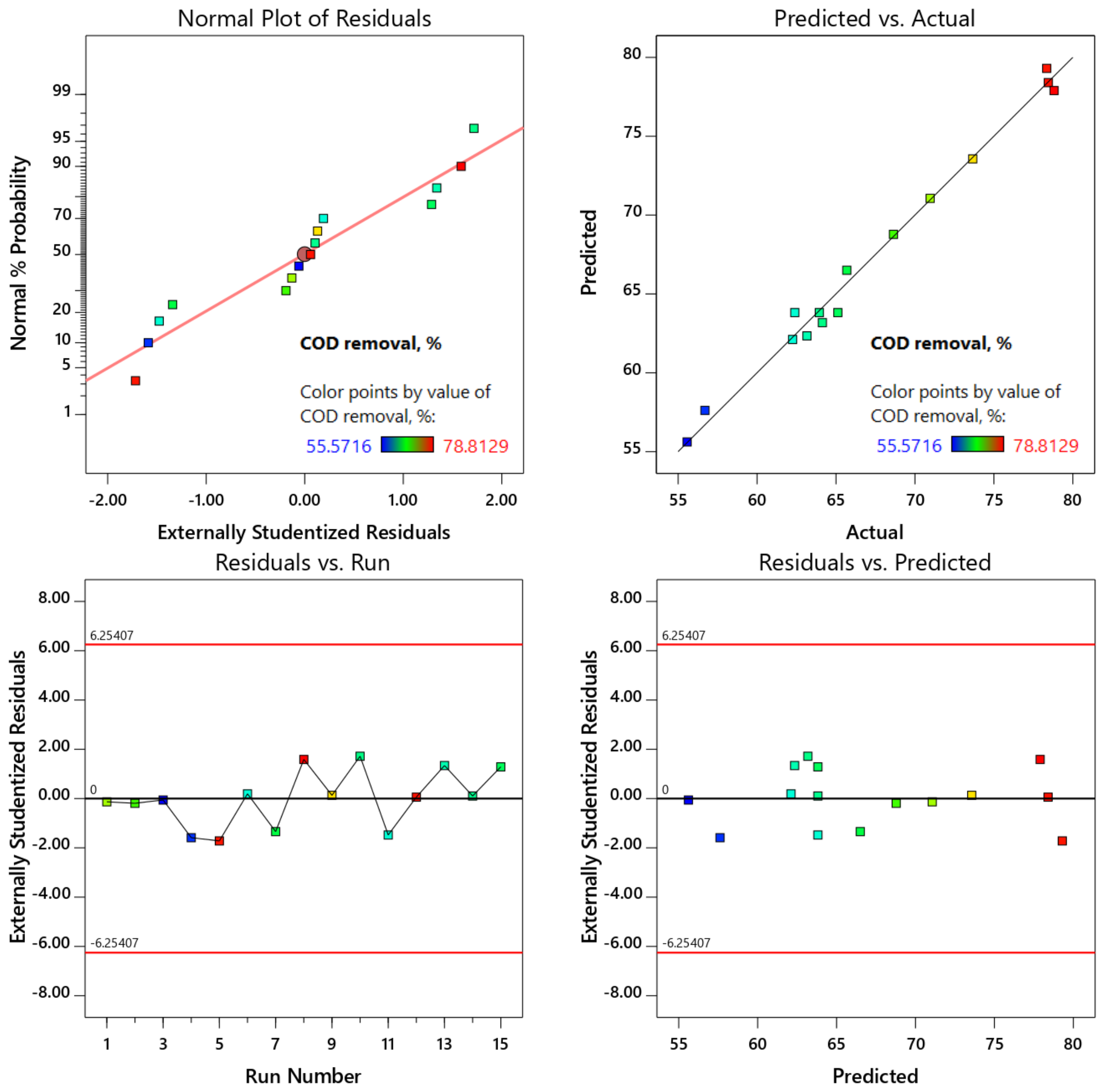

Figure S3. Parity and normal distribution plots for COD removal by the Co^{2+}/PAA process

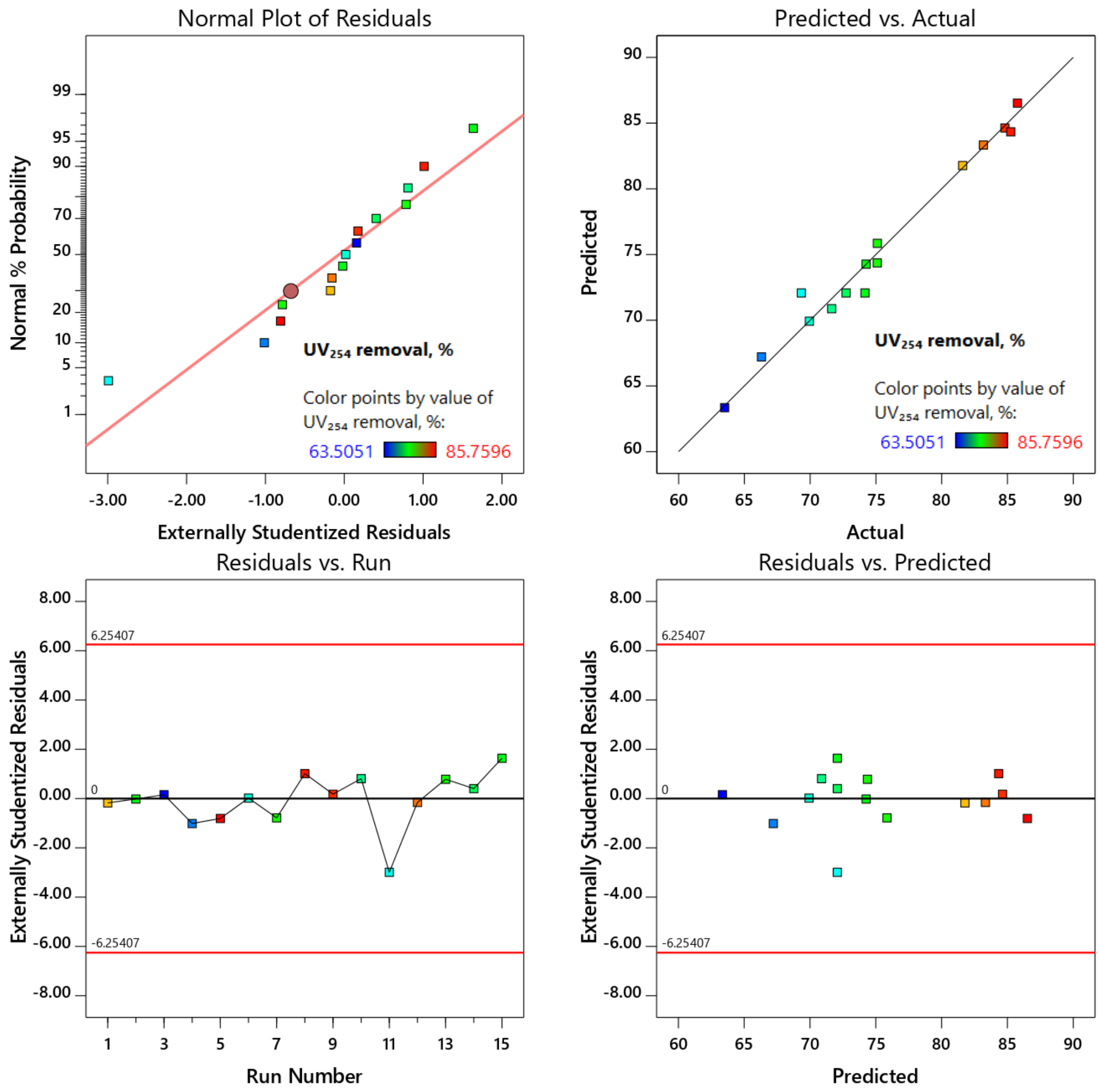

Figure S4. Parity and normal distribution plots for UV_{254} removal by the Co^{2+}/PAA process

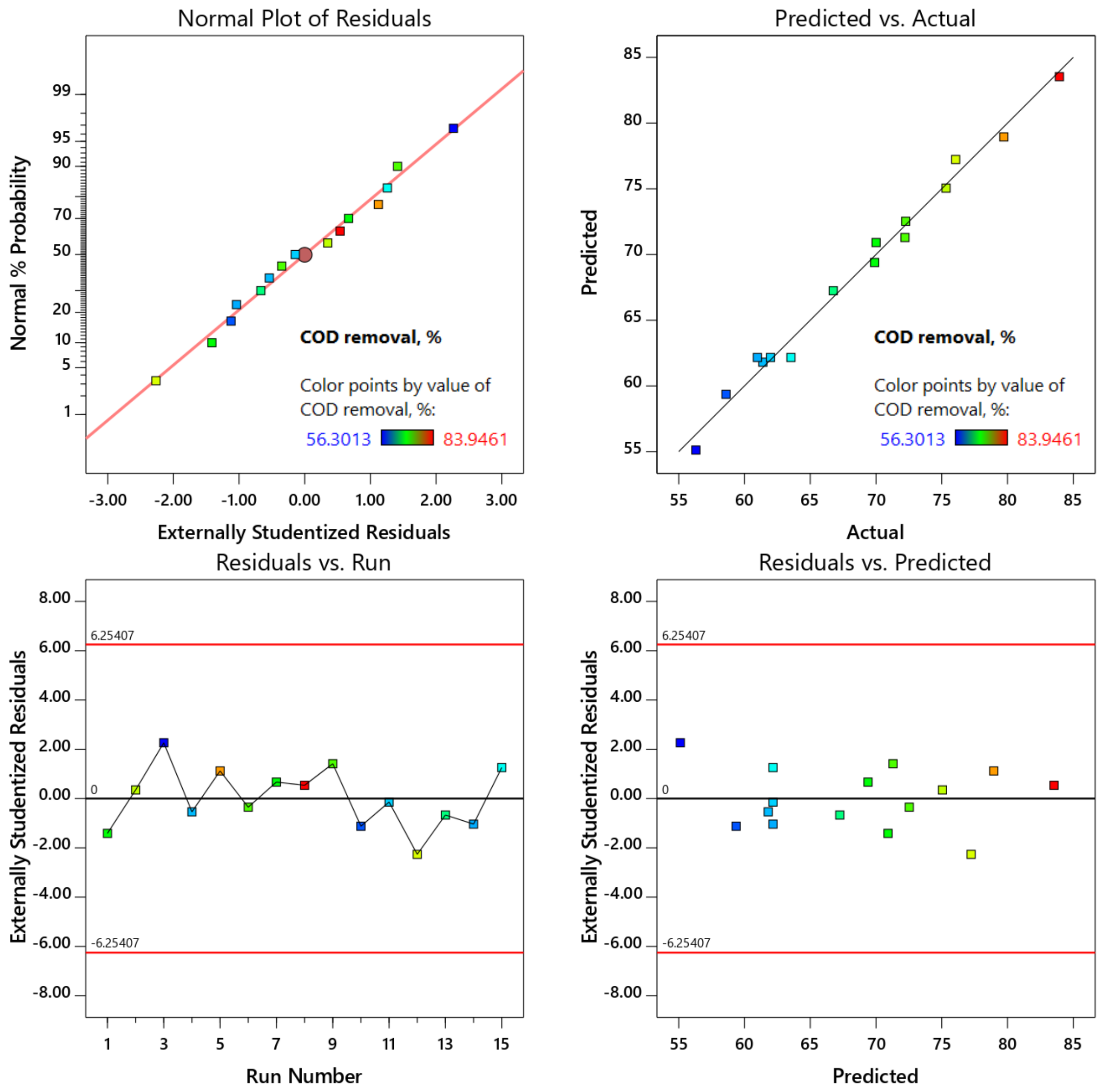

Figure S5. Parity and normal distribution plots for COD removal by the Mn^{2+}/PAA process

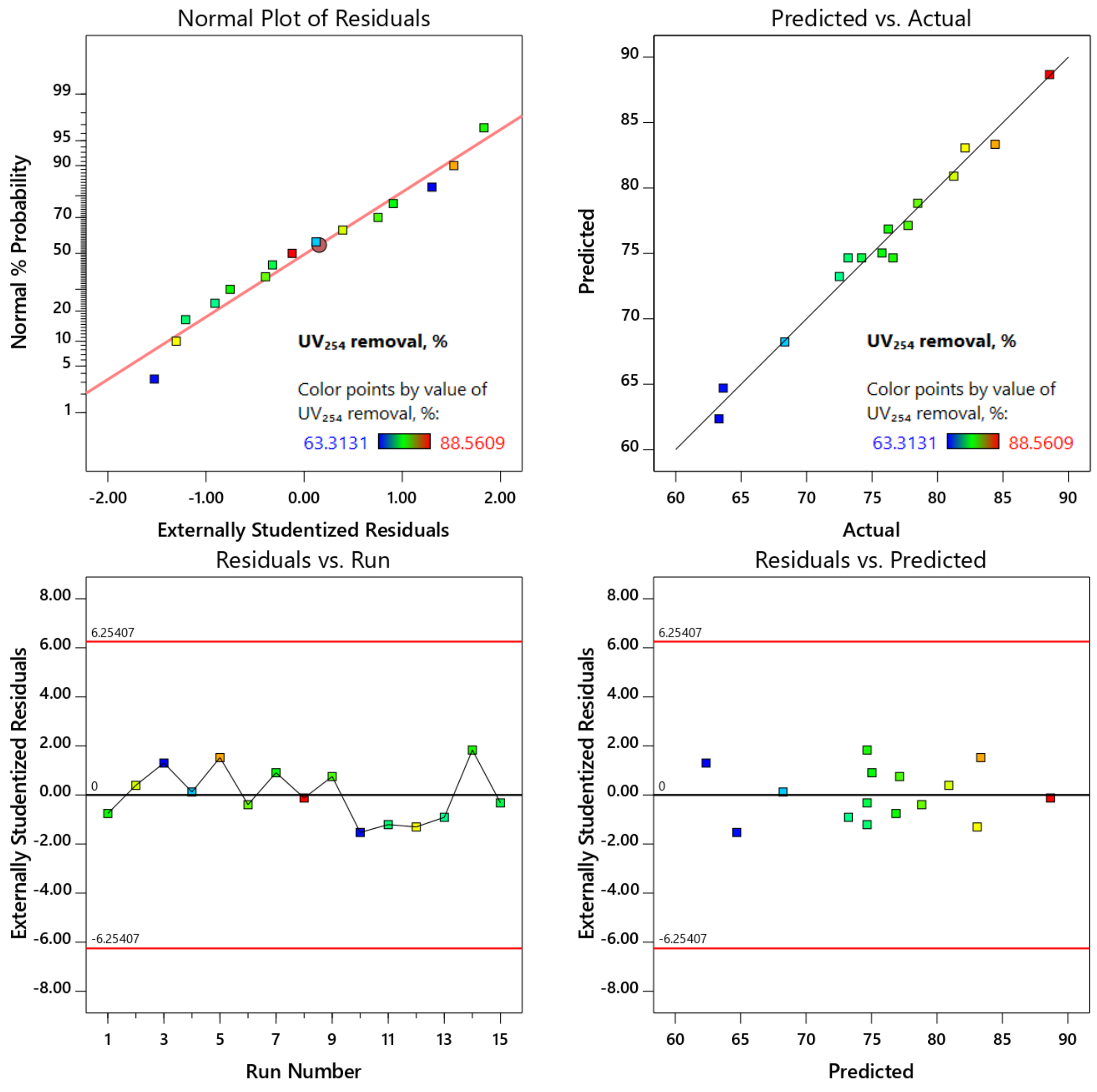

Figure S6. Parity and normal distribution plots for UV_{254} removal by the Mn^{2+}/PAA process

References

(1) Correa-Sanchez, S.; Peñuela, G. A. Peracetic Acid-Based Advanced Oxidation Processes for the Degradation of Emerging Pollutants: A Critical Review. Journal of Water Process Engineering 2022, 49, 102986.

(2) Zhao, Z.; Li, X.; Li, H.; Qian, J.; Pan, B. New Insights into the Activation of Peracetic Acid by Co (II): Role of Co (II)-Peracetic Acid Complex as the Dominant Intermediate Oxidant. ACS ES&T Engineering 2021, 1 (10), 1432–1440.

(3) Popov, E.; Eloranta, J.; Hietapelto, V.; Vuorenpalo, V.-M.; Aksela, R.; Jäkärä, J. Mechanism of Decomposition of Peracetic Acid by Manganese Ions and Diethylenetriaminepentaacetic Acid (DTPA). 2005.

(4) Kim, J.; Zhang, T.; Liu, W.; Du, P.; Dobson, J. T.; Huang, C.-H. Advanced Oxidation Process with Peracetic Acid and Fe (II) for Contaminant Degradation. Environ. Sci. Technol. 2019, 53 (22), 13312–13322.

(5) Kiejza, D.; Kotowska, U.; Polińska, W.; Karpińska, J. Peracids-New Oxidants in Advanced Oxidation Processes: The Use of Peracetic Acid, Peroxymonosulfate, and Persulfate Salts in the Removal of Organic Micropollutants of Emerging Concern− A Review. Science of the Total Environment 2021, 790, 148195.
